# OpenABC enables flexible, simplified, and efficient GPU accelerated simulations of biomolecular condensates

**DOI:** 10.1371/journal.pcbi.1011442

**Published:** 2023-09-11

**Authors:** Shuming Liu, Cong Wang, Andrew P. Latham, Xinqiang Ding, Bin Zhang

**Affiliations:** 1 Department of Chemistry, Massachusetts Institute of Technology, Cambridge, Massachusetts, United States of America; 2 Department of Bioengineering and Therapeutic Sciences, Department of Pharmaceutical Chemistry, Quantitative Biosciences Institute, University of California, San Francisco, San Francisco, California, United States of America; Fudan University, CHINA

## Abstract

Biomolecular condensates are important structures in various cellular processes but are challenging to study using traditional experimental techniques. In silico simulations with residue-level coarse-grained models strike a balance between computational efficiency and chemical accuracy. They could offer valuable insights by connecting the emergent properties of these complex systems with molecular sequences. However, existing coarse-grained models often lack easy-to-follow tutorials and are implemented in software that is not optimal for condensate simulations. To address these issues, we introduce OpenABC, a software package that greatly simplifies the setup and execution of coarse-grained condensate simulations with multiple force fields using Python scripting. OpenABC seamlessly integrates with the OpenMM molecular dynamics engine, enabling efficient simulations with performance on a single GPU that rivals the speed achieved by hundreds of CPUs. We also provide tools that convert coarse-grained configurations to all-atom structures for atomistic simulations. We anticipate that OpenABC will significantly facilitate the adoption of in silico simulations by a broader community to investigate the structural and dynamical properties of condensates.

## Author summary

Biomolecular condensates are essential cellular structures that underpin various cellular processes, including RNA splicing, ribosomal RNA processing, and stress response, etc. These membrane-less organelles exhibit liquid-like behavior, enriched with disordered proteins and RNA that can dynamically exchange with the surrounding media. Computational modeling aids in understanding these condensates’ structural and dynamic aspects at high resolution, connecting molecular sequences with condensate emergent properties. Both coarse-grained and atomistic simulations can help offer insights into the nature of the molecular interactions that drive phase separation, the microenvironment of the condensates, and their dynamical relaxation. However, software tools for such simulations are limited. We introduce OpenABC, a flexible software package for GPU-accelerated simulations of biomolecular condensates. It simplifies setup using Python scripts, integrates with the molecular dynamics engine, OpenMM, for efficient simulations, and facilitates transitioning between coarse-grained and atomistic representations. OpenABC aims to empower researchers by easing simulation setups, aiding force field comparison, and advancing biomolecular condensate research.

## Introduction

Biomolecular condensates underlie the organization of many cellular processes, such as speckles for RNA splicing, nucleoli for ribosomal RNA processes, and P granule for stress response, etc. [1–14]. They are also termed membrane-less organelles due to the lack of enclosure and exhibit liquid-like properties. Intrinsically disordered proteins (IDPs) and RNA molecules are enriched inside the condensates [3, 4, 9, 11]. These molecules promote promiscuous, multivalent interactions, leading to spontaneous phase transition and condensate formation [15]. The nature of the molecular interactions that drive phase separation, the microenvironment of the condensates, and their dynamical relaxation, are under active investigation.

Computational modeling can prove invaluable for studying biomolecular condensates by providing detailed structural and dynamic characterizations [16–42]. Particle-based coarse-grained modeling approaches are promising since their computational efficiency enables long-timescale simulations to promote large-scale reorganization for structural relaxation [43–46]. Such simulations may predict condensate physical properties de novo, elucidating the connection between molecular sequences and emergent properties [18, 47]. However, the reduced resolution of these coarse-grained models could be insufficient to describe the complex microenvironment of the condensate interior [48–50]. Atomistic simulations with explicit representation of solvent molecules and counter ions can be necessary to further characterize physicochemical interactions that produce the selective partition of small molecules within condensates [49–53]. Combining the two modeling approaches at different resolutions could be particularly powerful since they enable long-timescale simulations for structural relaxation while preserving the fine-resolution details.

While many computational models and force fields have been introduced for simulations of IDPs and biomolecules, software engineering has yet to catch up. There is an urgent need to build user-friendly tools to set up and execute condensate simulations. Preparing biomolecular simulations can be rather involved. Even creating initial configurations for such simulations is often non-trivial. Much-dedicated software has been introduced to prepare atomistic simulations [54–57], and existing molecular dynamics (MD) simulation packages are highly optimized for computational efficiency [54, 57–59]. However, existing tools are not immediately transferable for setting up coarse-grained condensate simulations. Furthermore, coarse-grained force fields are often implemented into disparate simulation engines not necessarily best suited for condensate simulations, hindering cross-validation and the unleashing of full modeling potential. Further software development can significantly reduce the entry barrier for in silico studies, allowing more researchers to experience the usefulness of computational modeling. They could facilitate comparing and benchmarking various force fields, driving continuous improvement.

We introduce a software package termed OpenABC for “**Open**MM GPU-**A**ccelerated simulations of **B**iomolecular **C**ondensates”. The package is flexible and implements multiple popular coarse-grained force fields for simulating proteins and nucleic acids. It dramatically simplifies the simulation setup: only a few lines of Python scripts are needed to carry out condensate simulations starting from initial configurations of a single protein or DNA. The package is integrated with OpenMM, a GPU-accelerated MD engine [60], enabling efficient simulations with advanced sampling techniques. Finally, we include tools that convert coarse-grained configurations to atomistic structures for further condensate modeling with all-atom force fields. Tutorials in Jupyter Notebooks are provided to demonstrate the various capabilities. We anticipate that OpenABC will greatly facilitate the application of existing computer models for simulating biomolecular condensates and the continued force field development.

## Results

### Flexible force field selections for Biomolecular simulations

**Fig 1:**
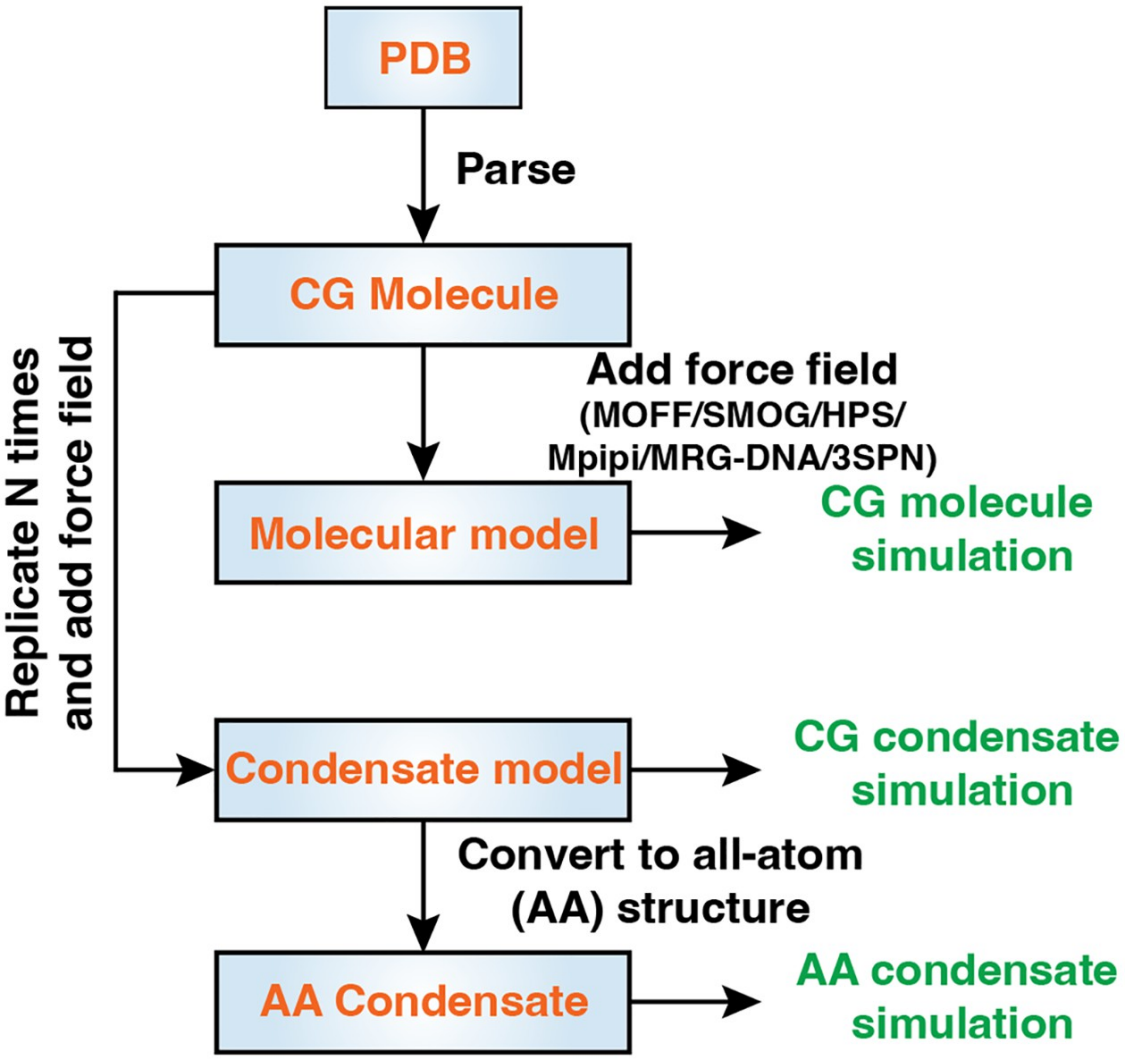
OpenABC facilitates coarse-grained and atomistic simulations of biomolecular condensates with multiple force fields. The diagram illustrates the workflow and various functionalities of OpenABC. To set up condensate simulations, the users must provide a configuration file in the PDB format for the molecule of interest. OpenABC parses topological and structural information from the PDB file to build a molecule object. Specifying force field options allows direct simulations of individual molecules. On the other hand, the molecule object can be replicated for condensate simulations. In addition, OpenABC allows the conversion of CG configurations to atomistic structures for simulations with all-atom force fields.

OpenABC implements several existing force fields for coarse-grained (CG) modeling of protein, RNA, and DNA molecules (Fig 1). Single-bead per amino acid force field for proteins include the hydropathy scale (HPS) models [20, 28], the Mpipi force field [29], a generalized structure-based model [41, 61, 62], and the maximum entropy optimized force field (MOFF) [63]. HPS models define interactions between different pairs of amino acids based on various hydrophobicity scales [20, 28]. Recent studies have attempted to improve the accuracy of HPS models with systematic optimizations of the hydrophobicity scale to match experimental observations of IDP monomers [34, 36]. They have been used to study the phase behaviors of numerous proteins [64–66], revealing the contribution of charge distribution patterns, cation-*π* interactions, and the balance between hydrophobic and electrostatic interactions [65, 67] to the stability of condensates.

The Mpipi force field was parameterized using data from all-atom simulations and bioinformatics analysis with a careful calibration of *π*-*π* and *π*-cation interactions [29]. These interactions play significant roles in the formation of biomolecular condensates. The force field was shown to accurately capture the radius of gyration and critical temperatures of diverse protein sequences.

SMOG was originally introduced for studying folded proteins using interaction potentials derived from initial input configurations. We generalized the model to describe proteins with disordered domains and leveraged the Miyazawa-Jernigan statistical potential [68] for protein-protein interactions [41, 62].

MOFF was parameterized with the maximum entropy algorithm [69, 70] and the protein folding energy landscape theory [71] to provide consistent descriptions of both folded and disordered proteins [63, 72–74]. It was shown to reproduce the radius of gyration for a collection of proteins, including both ordered and disordered proteins [63, 75]. The balanced interactions among amino acids have proven beneficial in describing complex contacts among phase-separating proteins, including those with both ordered and disordered domains [47, 63, 74].

In addition to protein models, we implemented several force fields for nucleic acids. For example, the molecular renormalization group coarse-graining (MRG-CG) DNA model was initially introduced for simulations with explicit ions to reproduce the salt-dependent DNA persistence length [76]. We adopted it for implicit ion modeling with the Debye-Hückel approximation for electrostatic interactions. We rescaled the strength of bonded interactions to ensure the accuracy of the implicit-ion model in reproducing DNA persistence length at the physiological salt concentration [47]. We further incorporated the DNA model 3SPN [77, 78] into OpenABC for studying sequence specific properties. Unlike MRG-CG DNA that only uses one bead to represent each nucleotide, 3SPN adopts three beads to differentiate sugar, base, and phosphate. Finally, the Mpipi force field can be used to simulate RNA molecules.

While one can in principle combine different force fields for simulating complex systems with both proteins and nucleic acids, care needs to be taken when modeling cross interactions. Previous studies have carried out systematic validations of protein-DNA and protein-RNA interactions and we implemented them into OpenABC, with combinations that include SMOG-3SPN [37, 41, 62, 78–82], MOFF-MRG-CG DNA [47], and Mpipi Protein-RNA [29]. These combinations account for both excluded volume effect and electrostatic interactions. Detailed expressions of all the force field potentials are provided in the S1 Text—*Force Field Definitions*, with the parameters provided in Tables A-I in S1 Text.

### Simplified setup of condensate simulations

OpenABC leverages the MD simulation engine, OpenMM [60], to offer simulation setup with Python scripting, thus dramatically simplifying the workflow. The software treats each molecule as an object and appends such objects into a container-like class. This class allows the incorporation of various force field options and integration schemes for MD simulations.

**Fig 2:**
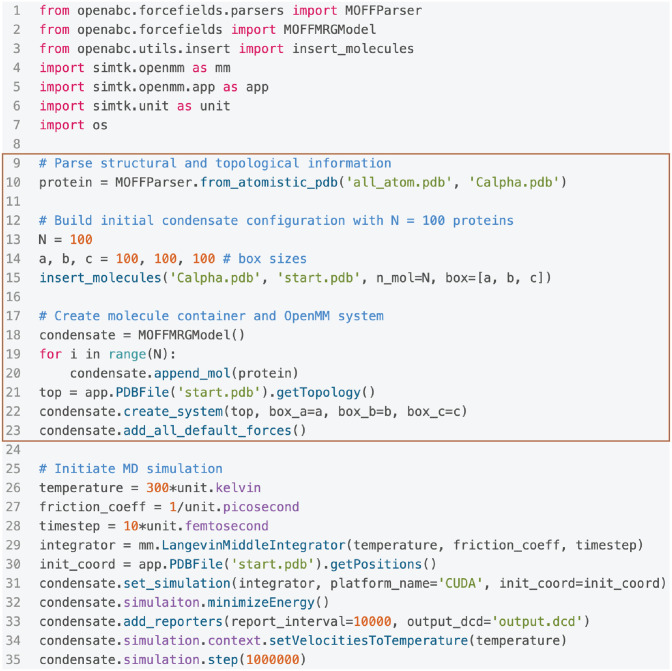
OpenABC simplifies simulation setup with Python scripting. The example code includes all the steps necessary for setting up and performing MD simulations of a protein condensate with MOFF and default settings in a cubic box of length 100 nm. The ten lines included in the highlight box correspond to the creation of the condensate system by parsing topological information from an initial PDB file, building a configuration file by inserting molecules into a box and incorporating the molecular objects, *protein*, into a container class, *condensate*, with appropriate force fields. The rest of the code includes standard simulation setups generic to OpenMM. We chose the Langevin middle integrator to perform simulations at 300 K with a friction coefficient of 1 ps^−1^ and a timestep of 10 fs.

An illustration of the typical workflow for condensate simulations is provided in Fig 1. OpenABC first parses a configuration file in the PDB format supplied by users to create a molecule object. The object contains topological and structural information extracted from the input file. Upon introducing interactions defined in various force fields, the molecule object can be used to simulate individual biomolecules. On the other hand, the molecule object can also be replicated *N* times for condensate simulations consisting of *N* molecules. As demonstrated in an example code in Fig 2, setting up an entire MD simulation of a protein condensate with default parameters only requires about 20 lines of code.

To enhance conformational sampling of individual molecules and condensates, we provide an implementation of the temperature replica exchange algorithm [83] with PyTorch [84] as part of the package (see S1 Text—*Implementation of the temperature replica exchange algorithm* for details). Furthermore, we introduce utility functions to reconstruct atomistic structures from coarse-grained protein configurations with only *α* carbons. This functionality relies on the software “reconstruct atomic model from reduced representation (REMO)” [85] and can facilitate downstream all-atom simulations. More tutorials in Jupyter Notebook format are available online at the OpenABC GitHub repository.

### Efficient simulations with GPU-enabled MD engine

A significant advantage of integrating with OpenMM comes from its native support of GPU acceleration. Simulating implicit solvent coarse-grained condensates on GPUs can be particularly beneficial due to the inhomogeneous distribution of particles arising from implicit solvation [78]. CPU parallelization, which often relies on the spatially-based, domain decomposition strategy, is often less effective because the inhomogeneity in particle density between the condensate and dilute phases produces an imbalanced workload between CPUs.

**Fig 3:**
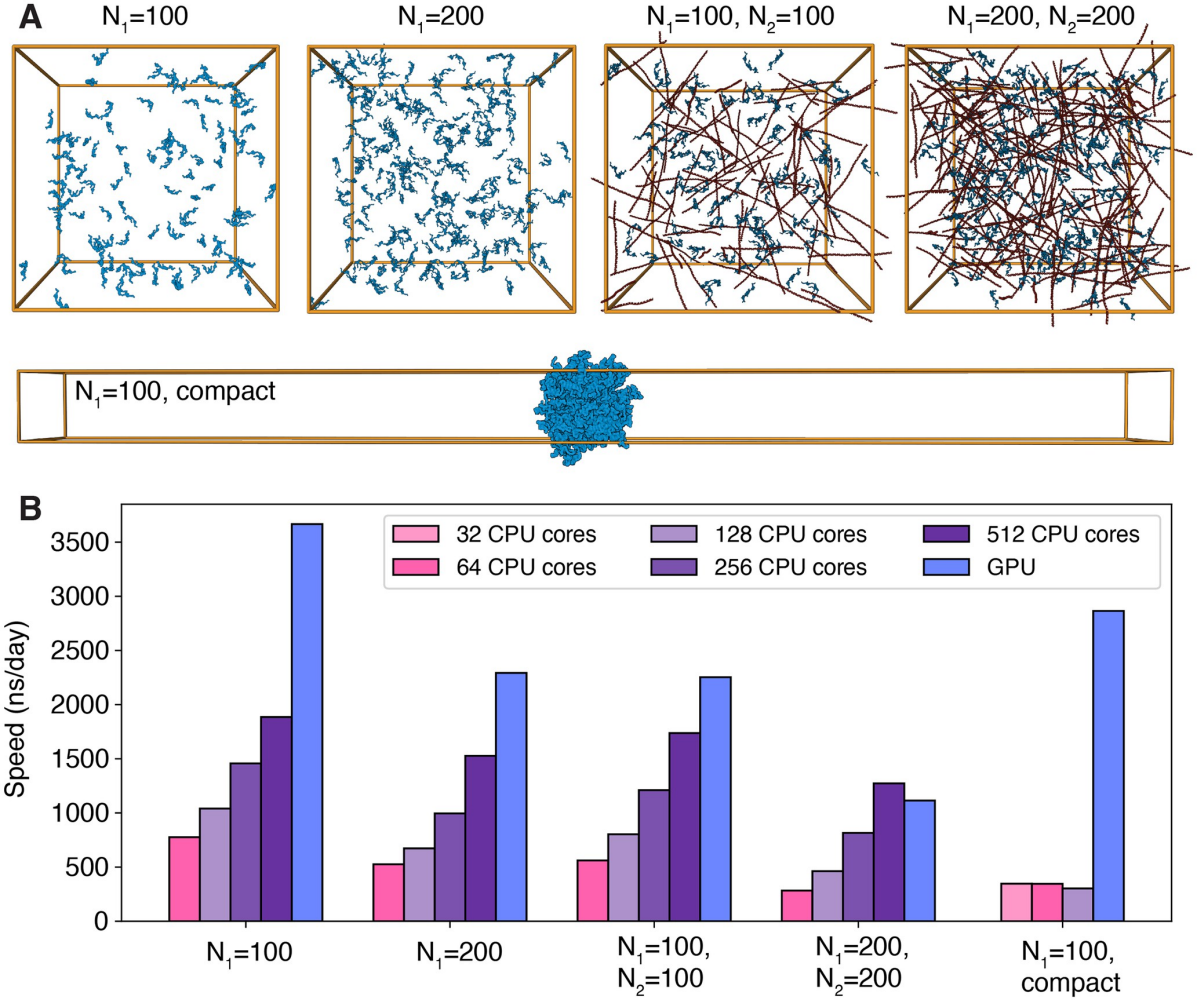
OpenABC integrates with OpenMM for GPU-accelerated MD simulations. (A) Snapshots of the five systems used to benchmark simulation performance. The systems consist of *N*_1_ HP1*α* dimers (blue) and *N*_2_ 200-bp-long dsDNA (red, *N*_2_ = 0 if not specified). The first four systems adopt homogeneous density distributions in cubic boxes of length 200 nm, while the last exhibits a dense-dilute interface in an elongated box of size 25 × 25 × 400 nm^3^. (B) The five data sets compare the performance of CPU simulations using GROMACS with single GPU simulations using OpenMM. The different colors indicate the number of CPUs in GROMACS simulations, as shown in the legends. The benchmarks were performed with Intel Xeon Gold 8260 CPUs and Nvidia Volta V100 GPUs.

To demonstrate the efficiency of GPU-enabled simulations, we studied five independent condensate systems. The first four systems consist of *N*_1_ HP1*α* dimers and *N*_2_ 200-bp-long dsDNA randomly distributed in a cubic box of length 200 nm with periodic boundary conditions. In the fifth system, 100 HP1*α* dimers in a compact configuration were placed at the center of an elongated box of size 25 × 25 × 400 nm^3^ (Fig 3A). This rectangular setup is typical for the so-called slab simulations to produce a dilute and dense interface along the *z*-axis for computing co-existence curves and phase diagrams [20, 86, 87]. MOFF and MRG-CG force fields were used to describe the interactions among coarse-grained particles. We simulated each system for one million steps using the Langevin middle integrator [88] to control the temperature at 300 K, with a friction coefficient of 1 ps^−1^ and a time step of 10 fs. For comparison, we simulated the same systems with a closely related integrator using GROMACS, a leading MD engine with state-of-the-art performance on CPUs [54, 57]. More simulation details are provided in the S1 Text—*Benchmarking the performance of condensate simulations*.

As shown in Fig 3B, OpenMM single GPU performance matches GROMACS with hundreds of CPUs in the first four systems. While GROMACS achieved nearly linear scaling for the first four systems, introducing more CPUs did not lead to any significant speedup in the last system with a dense-dilute interface. As mentioned above, the presence of vacuum regions in slab simulations hinders the efficacy of domain decomposition. On the other hand, OpenMM is less sensitive to the simulation setup and retains superior performance.

The performance of GROMACS depends on our implementation of the CG force fields and may not reflect the theoretical upper limit of the software. In particular, our use of tabulated potentials for the Debye Hückel potential and domain decomposition for parallelization may significantly affect the simulation speed. While performance improvement is possible with additional software engineering, the advantage of CG simulations of condensates on GPUs remains given the differences shown in Fig 3B.

### Application: Validating force field implementations in OpenABC

Before applying the software for extensive simulations, we validated our implementations of various force fields with existing ones. We generated ten configurations for an HP1*α* dimer with MOFF and GROMACS through an NVT simulation. As shown in Table J in S1 Text, the potential energies evaluated using MOFF from OpenABC match those reported by GROMACS. Similar comparisons with a protein-DNA complex produce nearly identical energy values as well, as shown in Table K in S1 Text. The protein-DNA complex is formed by an HP1*α* dimer with a 200-bp-long dsDNA, and MOFF and MRG-CG DNA were used to quantify their interactions. The minor differences between OpenMM and GROMACS energies are mainly caused by using tabulated functions for nonbonded interactions in GROMACS.

We further evaluated the potential energies defined by the HPS model on ten configurations of a disordered protein, DDX4, using both OpenMM and HOOMD-Blue [89]. As shown in Table L in S1 Text, the two sets of energies match exactly, supporting the correctness of our force field implementation. We also validated the Mpipi force field using interaction energies evaluated with OpenMM and LAMMPS [59] for a protein-RNA system, as shown in Table M in S1 Text.

**Fig 4:**
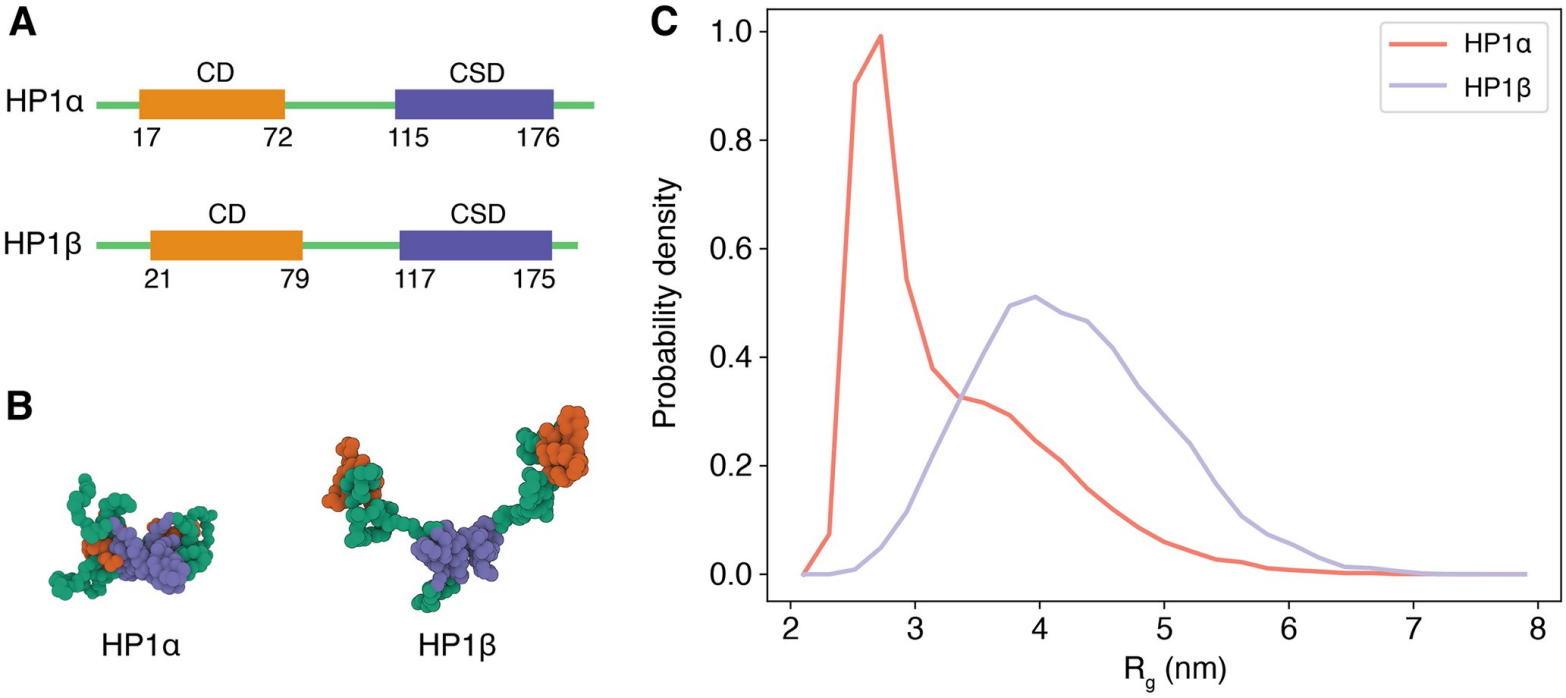
OpenABC produces consistent results with a previous studying, resolving the structural differences between two HP1 homologs. (A) Secondary structures of HP1*α* and HP1*β* along sequences. (B) Representative structures for HP1*α* and HP1*β* dimer rendered with Mol* Viewer [90]. The radii of gyration (*R*_*g*_) for the two structures are 2.77 and 4.44 nm, respectively. We colored the chromodomain (CD) in orange, the chromoshadow domain (CSD) in blue, and the rest in green. (C) Probability density distributions of *R*_*g*_ for HP1*α* (red) and HP1*β* dimer (blue).

In addition to energy comparisons, we examined the conformational ensembles of HP1*α* and HP1*β* dimers using MOFF with temperature replica exchange simulations [83]. Consistent with our previous study [63], the force field succeeds in resolving the difference in their conformational distribution between the two homologs (Fig 4). The radii of gyration for the two dimers at 300 K are 3.33 ± 0.19 nm, and 4.27 ± 0.09 nm, respectively. These values match the previously reported values computed using GROMACS quantitatively, reproducing experimental trends. Therefore, OpenABC produces consistent results with other software despite differences in integration schemes.

Using the MRG-DNA model, we computed the persistence length of a 200-bp-long DNA segment. The estimated value at a monovalent salt concentration of 100 mM, 48.83 ± 2.71 nm (see Fig A in S1 Text), is consistent with that reported in a previous study using simulations of the same model but with GROMACS [47]. Additional simulation details for estimating the persistence length are provided in the S1 Text—*Estimating the persistence length of MRG-DNA*.

### Application: Coarse-grained simulation of protein condensates

As additional evaluations of force field implementation and to demonstrate the usefulness of OpenABC, we performed slab simulations to determine the phase diagram of four proteins, which are known to form various biomolecular condensates inside the cell. For example, HP1 dimers are involved in chromatin compaction and regulation [91], while DDX4 and FUS are a primary constituent of nuage or germ granules [92] and cytoplasmic RNP granules [93], respectively. The simulations for HP1*α* and HP1*β* were performed with MOFF, while those for FUS LC and DDX4 were modeled with the HPS model using the shifted Urry hydrophobicity scale [28].

**Fig 5:**
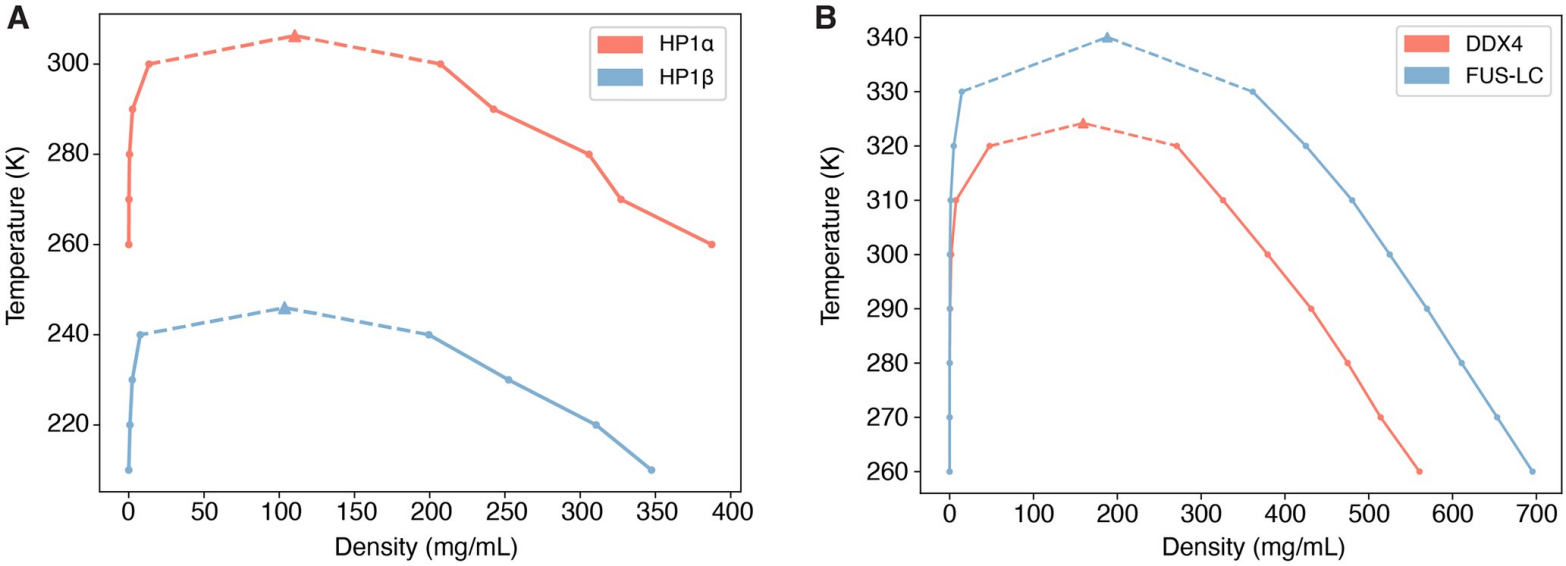
OpenABC produces phase diagrams that match previous results. (A) Phase diagrams for HP1*α* (red) and HP1*β* (blue) dimer condensates computed with MOFF. (B) Phase diagrams for DDX4 (red) and FUS LC (blue) computed with the HPS model parameterized using the Urry hydrophobicity scale. The dots in both plots denote the density values determined from slab simulations, and the triangles represent the critical point obtained from numerical fitting.

The resulting phase diagrams are shown in Fig 5, with the concentrations listed in Tables N-P in S1 Text. The density profiles at different temperatures and the representative snapshots at the lowest temperatures are shown in Figs B-C in S1 Text. We fitted the computed phase diagrams with an analytical expression to determine the critical temperature *T*_*c*_ (see Methods). The critical temperatures are 306.30 K for HP1*α* and 245.99 K for HP1*β*, consistent with previous results obtained with GROMACS simulations [63]. Similarly, the critical temperatures for DDX4 and FUS LC are 324.21 K and 340.04 K, respectively, matching values reported in a previous study that used the software HOOMD-Blue for simulations [28]. Thus, OpenABC produces statistically indistinguishable results on the phase behavior of protein condensates as in previous studies.

### Application: Atomistic simulation of protein condensates

While residue-level CG models are helpful for long timescale simulations, their limited resolution may prove insufficient to characterize specific properties of condensates, including the solvation environment [48], counter-ion distributions [49], and protein-ligand interactions [50]. Therefore, we implemented functionalities in OpenABC to convert equilibrated CG configurations to atomistic structures. Starting from these structures, well-established tools, such as CHARMM-GUI [55], GROMACS [54, 57], and AMBER [56], can be easily applied to set up explicit solvent simulations with diverse force fields. Furthermore, for explicit solvent simulations, the advantage of OpenMM over other MD packages is less evident. Therefore, we terminate the OpenABC workflow at producing atomistic condensate structures and leave the users with flexibility to choose MD packages and force fields for further studies.

**Fig 6:**
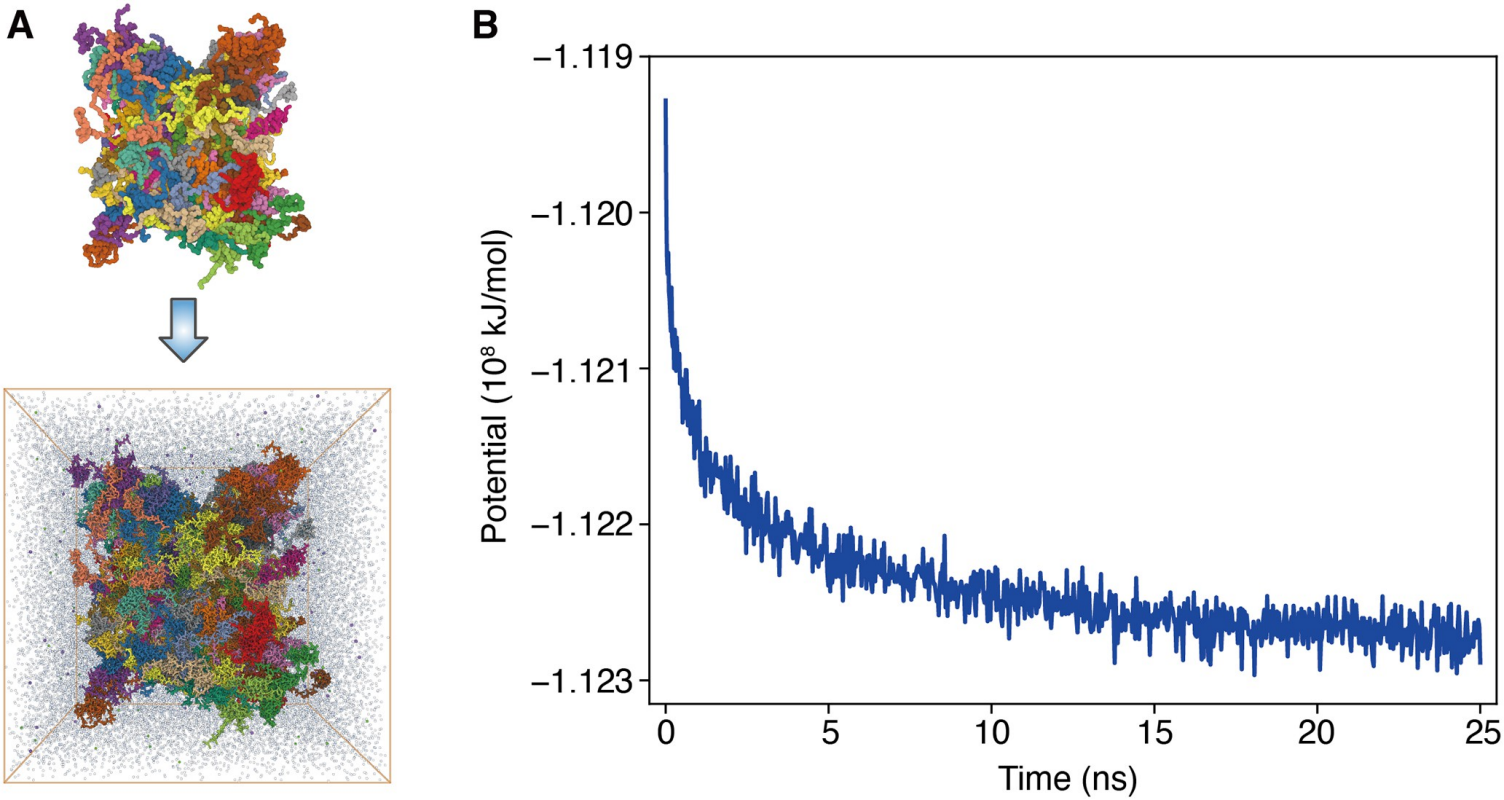
OpenABC facilitates all-atom simulations by producing equilibrated initial atomistic configurations. (A) Illustrations of the conversion from a coarse-grained configuration (top) to a fully atomistic model with explicit solvent molecules (bottom). Only 2% of water molecules and counter ions of the atomistic model are shown for clarity. The system consists of 100 HP1*α* dimers, and different molecules are shown in one of 25 colors. Both figures are rendered with Mol* Viewer [90]. (B) The atomistic potential energy evaluated using the CHARMM force field is shown as a function of simulation time.

As proof of principle, we converted the final snapshot from the slab simulation of HP1*α* dimer at 260 K to an atomistic configuration (Fig 6). This conversion leverages the software REMO [85] to build atomistic details starting from the C*α* positions of each amino acid. We solvated the atomistic HP1*α* dimer condensates with water molecules and counter ions. After energy minimization, we carried out an all-atom MD simulation using GROMACS with the CHARMM36m force field [94] and the CHARMM-modified TIP3P water model [95]. More details on simulation preparation can be found in the S1 Text—*Building and relaxing atomistic structures from coarse-grained configurations*. As shown in Fig 6, the system relaxes with a continuously decreasing potential energy in the first 20 ns and remains stable afterward.

## Conclusion

We introduced a software package, OpenABC, to facilitate coarse-grained and all-atom simulations of biomolecular condensates. The package implements several of the leading coarse-grained force fields for protein and DNA molecules into OpenMM, enabling GPU-accelerated simulations with performances rivaling GROMACS simulations with hundreds of CPUs. New force fields can be quickly introduced within the framework, and we plan to incorporate RNA models into the package as the next step. Comprehensive tutorials are provided to familiarize the users with the various functionalities offered by OpenABC. We anticipate the intuitive Python interface of OpenABC to reduce entry barriers and promote coarse-grained modeling for its adoption by a broader community.

## Materials and methods

### Details of molecular dynamics simulations

We performed temperature replica-exchange simulations [83] with MOFF to determine the conformational ensembles of HP1*α* and HP1*β* dimers. Atomistic protein structures were predicted with RaptorX [96] and used to initialize simulations. Details on modeling HP1 proteins to preserve the tertiary structure of folded domains are provided in the S1 Text—*Setting up MOFF HP1 system*. Six independent replicas were simulated to maintain temperatures at 300 K, 315.79 K, 333.33 K, 352.94 K, 375.00 K, and 400 K, respectively, with the Langevin middle integrator [88] and a friction coefficient of 1 ps^−1^. Each replica lasted for 200 million steps with a timestep of 10 fs. Exchanges between neighboring replicas were attempted every 1000 steps. More details on the replica exchange simulations are attached in the S1 Text—*Implementation of the temperature replica exchange algorithm*. We discarded the first 100 million steps as equilibration and used the remaining data for analysis.

We carried out slab simulations to evaluate the stability of condensates formed by HP1*α* and HP1*β* dimers. Initial configurations of these simulations were prepared as follows. First, we randomly placed 100 copies of protein dimers into a cubic box of length 75 nm. Then we performed 5-million-step constant pressure and constant temperature (NPT) simulations at one bar and 150 K to compress the system with a timestep of 10 fs. Control of pressure and temperature was achieved by coupling the Monte Carlo barostat with the Langevin middle integrator [88]. The length of the compressed cubic box was about 25 nm. Then we fixed the compressed configuration and extended the box size to 25 × 25 × 400 nm^3^. The rectangular geometry leads to the creation of a dense-dilute interface along the *z*-axis. Simulation results are expected to be independent of the exact box lengths and we chose 400 nm to be long enough to support phase coexistence (Fig B in S1 Text). Starting from this initial configuration, we gradually increased the temperature from 150 K to a target value in the first 0.1 million steps. We then performed 200-million-step production simulations at constant volume and constant temperature using the Nosé-Hoover integrator [88] with a collision frequency of 1 ps^−1^ and a timestep of 5 fs. Compared to the Langevin thermostat, the Nosé-Hoover integrator allows faster diffusion of protein molecules in the dilute phase to facilitate the equilibration of slab simulations.

Following similar protocols outlined above, we performed slab simulations for disordered regions of protein DDX4 and FUS with the HPS model using parameters derived from the Urry hydrophobicity scale [97]. Detailed amino acid sequences of the two proteins are provided in the S1 Text. For each protein, we first obtained an equilibrium configuration from a 0.1-million-step constant temperature simulation initialized with a straight C_*α*_ chain. We placed 100 replicas of the equilibrium configurations into a cubic box of length 75 nm. Upon compression by a 5-million-step NPT compression at 1 bar and 150 K with a timestep of 10 fs, the system reaches a cubic box with a size of about 15 nm. We then performed slab simulations with an elongated box of size 15 × 15 × 280 nm^3^ and a 10 fs timestep. Nosé-Hoover integrator was again applied with a collision frequency of 1 ps^−1^ to maintain the temperature.

### Computing phase diagrams from slab simulations

To determine the concentration of dense and dilute phases from slab simulations, we first identified the largest cluster in a given configuration as the largest connected component of the protein-contact network. Two monomers were defined as in contact if their center-of-mass distance was less than 5 nm, though the computed phase diagrams are rather insensitive to this specific cutoff value (Table O in S1 Text). Subsequently, we translated the system so that the center of mass of the largest cluster coincides with the box center, which was located at *z* = 0. We recognized the region with |*z*| < 5 nm for HPS simulations and |*z*| < 10 nm for MOFF simulations as the dense phase, while the region with |*z*| > 50 nm as the dilute phase. The threshold values were chosen to be consistent with prior literature [20, 63] and to roughly follow the size of the condensate as revealed in the density profiles (Figs B-C in S1 Text). The concentrations were determined as the average density value in specified regions using the second half of the simulation trajectories. We fitted the concentration values at various temperatures using the following equation to determine the critical temperature

$$\begin{array}{c} \rho_{\text{H}}-\rho_{\text{L}}=A{\left(T_{\text{c}}-T\right)}^{\beta }. \end{array}$$ (1)

*ρ*_H_ and *ρ*_L_ are the densities at the concentrated and dilute phases. Parameter *β* = 0.325 is the critical exponent corresponding to the universality class of 3D Ising model [98]. *T*_c_ is the critical temperature and *A* is the coefficient.

## Supporting information

S1 Text**Fig A**. The log of the bond vector correlation, log *C*(*n*), as a function of the bond separation *n*. The dots were obtained from MD simulations, with three colors indicate three independent simulations. The lines are numerical fits to the data. See text *Section: Computing DNA persistence length with the MRG-CG model* for simulation details and computing persistence length from the numerical fitting. **Fig B**. Density profiles obtained from slab simulations of HP1*α* (left) and HP1*β* (right) dimers with the MOFF model. Vertical lines are set at *z* = ±10 and ±50 nm. The final snapshots of the slab simulations at the lowest temperatures are shown. CG atoms with |*z*| < 10 nm are colored in yellow, while the remaining are shown in blue. **Fig C**. Density profiles obtained from slab simulations of DDX4 and FUS LC with the HPS model using the Urry scale optimal parameter set ($\mu =\mu_{\text{Urry}}^{\text{opt}}=1$ and $\Delta =\Delta_{\text{Urry}}^{\text{opt}}=0.08$) at different temperatures. Vertical dashed lines are set at *z* = ±5 nm and ±50 nm. The final snapshots of the slab simulations at 260 K are shown. CG atoms with |*z*| < 5 nm are colored in yellow, while the remaining are shown in blue. This figure shows that the |*z*| < 5 nm and |*z*| > 50 nm regimes can represent the concentrated and dilute phases, respectively. **Table A**. The amino acid mass, sizes, and charges used by MOFF and HPS models. Both models share the same amino acid mass and sizes. The charge of HIS differs in the two models, while other amino acids share the same charge. Here *e* is the elementary charge. **Table B**. MOFF protein contact *ϵ*_*ij*_ values as defined in equation S6 in S1 Text. Due to limited space, the numbers are rounded to 3 decimal places. The values are in unit kJ/mol. **Table C**. MRG DNA model bond parameters. All the *k*_bond,*n*_ are in unit kcal/mol/nm^2^, and *r*_0_ unit is nm. **Table D**. MRG DNA model angle parameters. All the *k*_angle,*n*_ are in unit kcal/mol/degree^2^, and *θ*_0_ unit is degree. **Table E**. MRG DNA model fan bond parameters. All the *k*_fan bond,*n*_ are in kcal/mol/nm^2^, and *r*_Δ,0_ unit is nm. Δ means the fan bond between CG nucleotide *i* and *j* + Δ, where nucleotide *i* and *j* are WC-paired. **Table F**. The normalized KR scale and Urry hydropathy scale values (i.e. λ_*i*_ parameters in equation S14 in S1 Text). **Table G**. Mpipi parameter *ϵ* values as defined in equation S25 in S1 Text. Due to limited space, the numbers are rounded to 3 decimal places. The values are in unit kJ/mol. **Table H**. Mpipi parameter *σ* values as defined in equation S25 in S1 Text. Due to limited space, the numbers are rounded to 3 decimal places. The values are in unit nm. **Table I**. SMOG MJ potential parameter *ϵ* as defined in equation S31 in S1 Text. Due to the limited space, the numbers are rounded to 3 decimal places. The values are in unit kJ/mol. **Table J**. Comparison of potential energies computed with OpenMM and GROMACS using MOFF for ten configurations of HP1*α* dimer. **Table K**. Comparison of potential energies computed with OpenMM and GROMACS using MOFF for proteins and MRG for DNA for ten configurations of HP1*α* dimer bound to a dsDNA. The energy unit is kJ/mol. See text *Section: Validating the force field implementation in OpenMM* for simulation details. **Table L**. Comparison of potential energies computed with OpenMM and HOOMD-Blue using HPS with Urry or KR scales for ten configurations of protein DDX4. The energy unit is kJ/mol. See text *Section: Validating the force field implementation in OpenMM* for simulation details. **Table M**. Comparison of potential energies computed with OpenMM and LAMMPS using Mpipi force field for a polyR+polyK+polyU system. The system consists of a chain of 10 arginines, a chain of 10 lysines, and 2 individual chains of 10 uracils. The energy unit is kJ/mol. See text *Section: Validating the force field implementation in OpenMM* for simulation details. **Table N**. The coexistence concentrations of HP1*α* and HP1*β* dimers measured by slab simulations with MOFF at different temperatures below the critical temperature. The cutoff distance for searching the largest cluster is 5 nm. **Table O**. The coexistence concentrations of HP1*α* and HP1*β* dimers measured by slab simulation with MOFF. The concentrations were similarly determined as those shown in Table N but the cutoff distance for searching the largest cluster set as 8 instead of 5 nm. The results are almost identical to the ones shown in Table N, supporting the robustness of phase diagrams with respect to the cutoff distance used for protein clustering. **Table P**. The coexistence concentrations of FUS LC and DDX4 proteins measured by performing slab simulations with HPS model Urry scale and the optimal parameter set ($\mu =\mu_{\text{Urry}}^{\text{opt}}=1$ and $\Delta =\Delta_{\text{Urry}}^{\text{opt}}=0.08$) at different temperatures below the critical temperature. The cutoff distance for searching the largest cluster is 5 nm.(PDF)
