# Supplementary material for: OpenABC enables flexible, simplified, and efficient GPU accelerated simulations of biomolecular condensates
Source: PLoS Comput Biol. 2023 Sep 11;19(9):e1011442. doi: 10.1371/journal.pcbi.1011442 (PMC10513381; doi:10.1371/journal.pcbi.1011442)
Supplement: S1 Text — Fig A. The log of the bond vector correlation, log C(n), as a function of the bond separation n. The dots were obtained from MD simulations, with three colors indicate three independent simulations. The lines are numerical fits to the data. See text Section: Computing DNA persistence length with the MRG-CG model for simulation details and computing persistence length from the numerical fitting. Fig B. Density profiles obtained from slab simulations of HP1α (left) and HP1β (right) dimers with the MOFF model. Vertical lines are set at z = ±10 and ±50 nm. The final snapshots of the slab simulations at the lowest temperatures are shown. CG atoms with |z| < 10 nm are colored in yellow, while the remaining are shown in blue. Fig C. Density profiles obtained from slab simulations of DDX4 and FUS LC with the HPS model using the Urry scale optimal parameter set (μ=μUrryopt=1 and Δ=ΔUrryopt=0.08) at different temperatures. Vertical dashed lines are set at z = ±5 nm and ±50 nm. The final snapshots of the slab simulations at 260 K are shown. CG atoms with |z| < 5 nm are colored in yellow, while the remaining are shown in blue. This figure shows that the |z| < 5 nm and |z| > 50 nm regimes can represent the concentrated and dilute phases, respectively. Table A. The amino acid mass, sizes, and charges used by MOFF and HPS models. Both models share the same amino acid mass and sizes. The charge of HIS differs in the two models, while other amino acids share the same charge. Here e is the elementary charge. Table B. MOFF protein contact ϵij values as defined in equation S6 in S1 Text. Due to limited space, the numbers are rounded to 3 decimal places. The values are in unit kJ/mol. Table C. MRG DNA model bond parameters. All the kbond,n are in unit kcal/mol/nm2, and r0 unit is nm. Table D. MRG DNA model angle parameters. All the kangle,n are in unit kcal/mol/degree2, and θ0 unit is degree. Table E. MRG DNA model fan bond parameters. All the kfan bond,n are in kcal/mol/nm2, and rΔ,0 u [file pcbi.1011442.s001.pdf]

Supporting Information: OpenABC Enables Flexible, Simplified, and Efficient GPU Accelerated Simulations of Biomolecular Condensates

Shuming Liu[1], Cong Wang[1], Andrew P. Latham[1, 2], Xinqiang Ding[1], and Bin Zhang[1*]

E-mail: binz@mit.edu

1 Department of Chemistry, Massachusetts Institute of Technology, Cambridge, Massachusetts, United States of America

2 Department of Bioengineering and Therapeutic Sciences, Department of Pharmaceutical Chemistry, Quantitative Biosciences Institute, University of California, San Francisco, San Francisco, California, United States of America

Contents

Force Field Definitions **S-3**

MOFF protein force field . . . S-3

HPS protein force field . . . S-6

MRG-CG DNA force field . . . S-7

MOFF Protein-MRG DNA interactions . . . S-9

Mpipi protein and RNA force field . . . S-9

The generalized structure-based protein model . . . S-10

SMOG Protein-3SPN2 DNA interactions . . . S-12

Details for Preparing and Performing Simulations **S-13**

Python implementation of the shadow algorithm for native pair identification . . . S-13

Implementation of the temperature replica exchange algorithm . . . S-16

Building and relaxing atomistic structures from coarse-grained configurations . . . S-16

Setting up MOFF HP1 system . . . S-17

Benchmarking the performance of condensate simulations . . . S-18

Validating the force field implementation in OpenMM . . . S-19

Computing DNA persistence length with the MRG-CG model . . . S-20

Sequences **S-21**

HP1α monomer . . . S-21

HP1β monomer . . . S-22

FUS LC . . . S-22

DDX4 . . . S-22

Reference **S-40**

Force Field Definitions

MOFF protein force field

MOFF is a transferable protein force field optimized for both ordered and disordered proteins utilizing the maximum entropy principle and ordered protein folding landscape.[S1] Each amino acid is represented with one coarse-grained (CG) bead whose position is defined using the C_α atom from the atomistic structures. MOFF energy function is defined as

$$U_{\text{MOFF}} = U_{\text{bond}} + U_{\text{angle}} + U_{\text{dihedral}} + U_{\text{memory}} + U_{\text{contact}} + U_{\text{electrostatics}} \tag{S1}$$

The bonded term, U_{bond}, consists of harmonic potentials for distances $r_{i,i+1}$ between bonded nearest neighbor beads

$$U_{\text{bond}} = \sum_i \frac{1}{2} k_{\text{bond}} (r_{i,i+1} - r_0)^2, \tag{S2}$$

where the equilibrium length $r_0 = 0.38$ nm, and force constant $k_{\text{bond}} = 1000$ kJ/mol/nm^2.

The angular term, U_{angle}, consists of harmonic potentials for angles between nearest neighbor bonds

$$U_{\text{angle}} = \sum_i \frac{1}{2} k_{\text{angle}} (\theta_i - \theta_{i,0})^2, \tag{S3}$$

where θ_i is the i-th angle. The equilibrium angles, $\theta_{i,0}$, are measured from the input atomistic structure and force constant $k_{\text{angle}} = 120$ kJ/mol/rad^2.

The dihedral term, U_{dihedral}, consists of periodic torsion potentials with periodicity $n = 1$ or 3

$$U_{\text{dihedral}} = \sum_i \sum_{n=1,3} k_{\text{dihedral},n} [1 + \cos(n(\theta_i - \theta_{i,0} - \pi))] \tag{S4}$$

where θ_i is the i-th dihedral. The equilibrium values, $\theta_{i,0}$, are measured from the input

atomistic structure, $k_{\text{dihedral},1} = 3.0$ kJ/mol/rad^2, and $k_{\text{dihedral},3} = 1.5$ kJ/mol/rad^2.

The memory potential, U_{memory}, also known as native pair potential, stabilizes the ordered secondary and tertiary structures of folded protein domains. It is limited to native pairs identified from the initial input native structure using the Shadow Algorithm detailed below. It is defined as

$$U_{\text{memory}} = \sum_{\substack{\text{native pairs } (i,j) \\ i<j}} \epsilon \left[5\left(\frac{\mu_{ij}}{r_{ij}}\right)^{12} - 6\left(\frac{\mu_{ij}}{r_{ij}}\right)^{10} \right]. \tag{S5}$$

μ_{ij} is the distance between Cα atoms from residue i and j measured from the input native structure, and by default $\epsilon = 3$ kJ/mol.

The contact potential, U_{contact}, measures nonbonded interactions between pairs of amino acids. It involves a repulsion term for excluded volume effect and a specific term that measures the energetic cost of bringing a pair of amino acids into contact. The expression is

$$U_{\text{contact}} = \sum_{i<j} \left\{ \frac{\alpha_{ij}}{r^{12}} - \frac{\epsilon_{ij}}{2}[1 + \tanh(\eta(r_0 - r))] \right\} \tag{S6}$$

$\eta = 7$ nm^{-1} and $r_0 = 0.8$ nm. α_{ij} and ϵ_{ij} are parameters depending on amino acid type i and j. $\alpha_{ij} = \sigma_{ij}^{12}|\epsilon_{ij}|$. $\sigma_{ij} = (\sigma_i + \sigma_j)/2$ where σ_i is the size of amino acid i. The amino acid sizes (i.e. σ_i) are listed in A, and parameters ϵ_{ij} are listed in B. We used a cutoff distance $r_c = 2$ nm, and the potential is shifted to zero and remains continuous at $r = r_c$ (such shift is not explicitly shown in equation S6). 1-2, 1-3, 1-4 atom pairs, and native pairs are excluded from the sum.

The electrostatic potential, $U_{\text{electrostatics}}$, is defined with the Debye-Hückel potential with a distance-dependent relative permittivity (dielectric) $\epsilon(r)$ as

$$U_{\text{electrostatics}} = \sum_{i<j} \frac{S(r_{ij})Q_iQ_j}{4\pi\epsilon_0\epsilon(r_{ij})r_{ij}} \exp(-r_{ij}/\lambda_D(r_{ij})), \tag{S7}$$

with

$$\epsilon(r) = A + \frac{B}{1 + \kappa \exp(-\zeta B r)}, \tag{S8}$$

and

$$\lambda_D(r) = \sqrt{\frac{k_B T \epsilon_0 \epsilon(r)}{2 N_A c e^2}}. \tag{S9}$$

Q_i and Q_j denote the charges of residues i and j with values provided in A, and ϵ_0 is the vacuum permittivity. 1-2, 1-3, and 1-4 pairs are excluded from the sum, but native pairs are included. The distance-dependent dielectric, $\epsilon(r)$, switches continuously between the value in water (ϵ_{water}) to the one in bulk protein, with $A = -8.5525$, $B = \epsilon_{\text{water}} - A$, $\kappa = 7.7839$, and $\zeta = 0.03627$ nm^{-1}. $\epsilon_{\text{water}} = 78.4$ is the relative permittivity (dielectric) constant of water. λ_D is the Debye length, which also depends on distance. The variables in equation S9 correspond to the Boltzmann constant (k_B), temperature (T), the Avogadro constant (N_A), the monovalent salt concentration (c), and the elementary charge (i.e. proton charge) (e), respectively.

The switch function gradually turns off the electrostatic interaction within regime $r_1 \leq r \leq r_2$

$$S(r) = \begin{cases} 1 & (r < r_1) \\ \sum_{i=0}^{5} a_n \left(\frac{r - r_1}{r_2 - r_1} \right)^n & (r_1 \leq r < r_2)\,, \\ 0 & (r \geq r_2) \end{cases} \tag{S10}$$

where $a_0, a_1, \cdots, a_5$ equal 1, 0, 0, -10, 15, and -6, respectively. Thus the switch function equals 1 at $r_1 = 1.2$ nm while 0 at $r_2 = 1.5$ nm.

HPS protein force field

Hydropathy scale (HPS) models[S2,S3] are designed to simulate the phase behaviors of intrinsically disordered proteins (IDP). The energy function of these models is defined as

$$U_{\text{HPS}} = U_{\text{bond}} + U_{\text{electrostatics}} + U_{\text{AH}}. \tag{S11}$$

The bonded term, U_{bond}, consists of harmonic potentials for distances $r_{i,i+1}$ between nearest neighbor beads

$$U_{\text{bond}} = \sum_i \frac{1}{2} k_{\text{bond}} (r_{i,i+1} - r_0)^2, \tag{S12}$$

where the equilibrium length $r_0 = 0.38$ nm, and force constant $k_{\text{bond}} = 8368$ kJ/mol/nm^2.

The electrostatic potential, $U_{\text{electrostatics}}$, is defined with the Debye-Hückel potential as

$$U_{\text{electrostatics}} = \sum_{i<j} \frac{Q_i Q_j}{4\pi\epsilon_0 \epsilon r} \exp(-r_{ij}/\lambda_D), \tag{S13}$$

where ϵ_0 is the vaccum permittivity, $\epsilon = 80$ is the relative permittivity of water, and $\lambda_D = 1$ nm is Debye length at the monovalent salt concentration of 100 mM. Q_i and Q_j denote the charges of residues i and j with values provided in A.

The AH potential describes amino acid-specific interactions using Ashbaugh and Hatch's functional form[S4] as

$$U_{\text{AH}} = \sum_{i<j} \begin{cases} [\phi_{ij}^{\text{LJ}}(r_{ij}) + (1 - \lambda_{ij})\epsilon] & r_{ij} \leq 2^{1/6}\sigma_{ij} \\ \lambda_{ij}\phi_{ij}^{\text{LJ}}(r_{ij}) & r_{ij} > 2^{1/6}\sigma_{ij}. \end{cases} \tag{S14}$$

$\lambda_{ij} = \mu\lambda_{ij}^0 - \Delta$ and $\lambda_{ij}^0 = (\lambda_i + \lambda_j)/2$, where λ_i is the normalized hydropathy scale of amino acid i. μ and Δ are the scaling and drift factors, respectively. λ_i represents normalized hydrophobicity scales. By default, the scale by Kapcha and Rossky (KR scale)[S5] is applied with $\mu_{\text{KR}} = 1$, $\Delta_{\text{KR}} = 0.0$ and the scale by Urry et al. (Urry scale)[S6] is applied with $\mu_{\text{Urry}} = 1$,

$\Delta_{\text{Urry}} = 0.08$. These are the optimal scaling and drift factors for the two hydropathy scales.[S3]

ϕ_{ij}^{LJ} in equation S14 corresponds to the normal Lennard-Jones potential defined as

$$\phi_{ij}^{\text{LJ}}(r) = 4\epsilon \left[\left(\frac{\sigma_{ij}}{r} \right)^{12} - \left(\frac{\sigma_{ij}}{r} \right)^{6} \right], \tag{S15}$$

with $\epsilon = 0.8368$ kJ/mol. $\sigma_{ij} = (\sigma_i + \sigma_j)/2$, where σ_i is the size of amino acid i with values provided in A.

We use a cutoff distance of 3.5 nm for electrostatic interactions and $4\sigma_{ij}$ for the amino-acid-specific AH potential. Both potentials were shifted by the values at the cutoff to ensure continuity (the shifts are not explicitly shown in equation S13 and S14). Furthermore, nearest neighbors (i.e. bonded atom pairs) are excluded from the electrostatic and AH potentials.

MRG-CG DNA force field

MRG-CG DNA force field was originally developed by Savelyev et al for simulating Watson–Crick (WC) paired dsDNA with the explicit presence of counter ions.[S7] Each nucleotide is represented with one CG bead of mass 325 Da. The energy function excluding ions is defined as

$$U_{\text{DNA}} = U_{\text{bond}} + U_{\text{angle}} + U_{\text{fan-bond}} + U_{\text{contact}} + U_{\text{electrostatics}} \tag{S16}$$

Bonded potentials are applied to every two neighboring CG atoms on each ssDNA chain, and

$$U_{\text{bond}} = \sum_i \sum_{n=2}^{4} k_{\text{bond},n} (r_{i,i+1} - r_0)^n. \tag{S17}$$

Values for the spring constants, $k_{\text{bond},n}$, and the equilibrium distance, r_0, are listed in C.

Angular potentials are applied to every three neighboring CG atoms on each ssDNA chain, and

$$U_{\text{angle}} = \sum_i \sum_{n=2}^{4} k_{\text{angle},n} (\theta_i - \theta_0)^n. \tag{S18}$$

θ_i is the i-th angle. Values for the spring constants, $k_{\text{angle},n}$, and the equilibrium distance,

θ_0, are listed in C.

The fan bonds are introduced to capture base-pairing, cross-stacking, and other interactions between two ssDNA chains. They are applied between CG beads i and $j-5, j-4, \cdots, j+5$, where bead i and j form a WC pair.

$$U_{\text{fan-bond}} = \sum_{\substack{\text{WC pair }(i,j)\\ i<j}} \sum_{\Delta=-5}^{5} \sum_{n=2}^{4} k_{\text{fan bond},n} (r_{i,j+\Delta} - r_{\Delta,0})^n \tag{S19}$$

Values for the spring constants, $k_{\text{fan bond},n}$, and the equilibrium distance, $r_{\Delta,0}$, are listed in E.

To adapt the force field to implicit-ion simulations, we scaled the bonded interactions by a factor of 0.9 so that the simulated persistence length for dsDNA matches the experimental value.[S1] Note the parameter values listed in C, D, and E are the original values reported in reference S7 before scaling. We further replaced the original electrostatic interaction with the same Debye-Hückel electrostatic interaction with distance-dependent dielectric defined in equation S7. Each CG nucleotide atom possesses a $-e$ charge.

Finally, the nonbonded contact potential for excluded volume effect is defined as

$$U_{\text{contact}} = \sum_{i<j} \frac{\alpha_{\text{DNA-DNA}}}{r_{ij}^{12}}, \tag{S20}$$

with $\alpha_{\text{DNA-DNA}}$ set as $1.678 \times 10^{-5}\ \text{nm}^{12} \cdot \text{kJ/mol}$.

Nearest neighbor CG beads with bonded and angular potentials are excluded from contact and electrostatic interactions. CG beads with fan bonds are from different DNA strands and are not excluded from such nonbonded interactions.

MOFF Protein-MRG DNA interactions

MOFF Protein-MRG DNA interactions include both contact and electrostatic potentials. The contact potentials are defined as

$$U_{\text{contact}} = \sum_{i<j} \frac{\alpha_{\text{protein-DNA}}}{r_{ij}^{12}}, \tag{S21}$$

with $\alpha_{\text{protein-DNA}} = 1.6264 \times 10^{-3}\ \text{nm}^{12} \cdot \text{kJ/mol}$. Electrostatic interactions follow the same definition as in equation S7. A previous study has shown that the above simple treatment of protein-DNA interactions successfully reproduces the binding free energy of several complexes.[S8]

Mpipi protein and RNA force field

The Mpipi force field is developed based on atomistic simulations and bioinformatic data.[S9] Its energy function is defined as

$$U_{\text{Mpipi}} = U_{\text{bond}} + U_{\text{electrostatics}} + U_{\text{WF}} \tag{S22}$$

The bond term U_{bond} is of the harmonic form

$$U_{\text{bond}} = \sum_{i} \frac{1}{2} k_{\text{bond}} (r_{i,i+1} - r_0)^2 \tag{S23}$$

with $k_{\text{bond}} = 1920\ \text{kcal/mol/nm}^2$. r_0 is 0.381 nm for protein, and 0.5 nm for RNA molecules.

The electrostatic interaction is computed with the Debye-Hückel potential

$$U_{\text{electrostatics}} = \sum_{i<j} \frac{Q_i Q_j}{4\pi\epsilon_0\epsilon r} \exp(-r_{ij}/\lambda_D) \tag{S24}$$

with $\epsilon = 80$ for water. The Debye length λ_D depends on the salt concentration of the system. Notably, the model uses scaled charges for amino acids and nucleotides, with $+0.75e$ for Arg

and Lys, $-0.75e$ for Asp and Glu, $+0.375e$ for His, and $-0.75e$ for nucleotides.

Nonbonded interactions are captured by the Wang-Frenkel potential[S10]

$$\begin{aligned} U_{\mathrm{WF}} =& \sum_{i<j} \epsilon_{ij}\alpha_{ij} \left[\left(\frac{\sigma_{ij}}{r}\right)^{2\mu_{ij}} - 1\right] \left[\left(\frac{R_{ij}}{r}\right)^{2\mu_{ij}} - 1\right]^{2\nu_{ij}} \\ \alpha_{ij} =& 2\nu_{ij} \left(\frac{R_{ij}}{\sigma_{ij}}\right)^{2\mu_{ij}} \left\{ \frac{2\nu_{ij}+1}{2\nu_{ij}\left[\left(\frac{R_{ij}}{\sigma_{ij}}\right)^{2\mu_{ij}} - 1\right]} \right\}^{2\nu_{ij}+1} \end{aligned} \tag{S25}$$

with cutoff at $R_{ij} = 3\sigma_{ij}$. All the ϵ_{ij} and σ_{ij} values are listed in G and H. μ_{ij} for most pairs is set as 2, except for two Ile as 11, for Val and Ile as 4, and for protein and RNA or RNA and RNA as 3. All the ν_{ij} values are equal to 1.

The generalized structure-based protein model

The structure-based model (SMOG) was originally introduced for modeling single folded proteins. It requires an input configuration file with the protein in the native state to define the stabilizing interaction potential. The SMOG energy function is defined as

$$U_{\mathrm{SMOG}} = U_{\mathrm{bond}} + U_{\mathrm{angle}} + U_{\mathrm{dihedral}} + U_{\mathrm{memory}} + U_{\mathrm{contact}} + U_{\mathrm{electrostatics}} \tag{S26}$$

The bonded term, U_{bond} is defined using harmonic potentials

$$U_{\mathrm{bond}} = \sum_i \frac{1}{2} k_{\mathrm{bond}} (r_{i,i+1} - r^o_{i,i+1})^2, \tag{S27}$$

where $r_{i,i+1}$ is the distance between two Cα atoms. $r^o_{i,i+1}$ is the corresponding value measured from the input native structure. By default $k_{\mathrm{bond}} = 50000$ kJ/mol/nm^2.

The angle term, U_{angle}, is defined as

$$U_{\mathrm{angle}} = \sum_i \frac{1}{2} k_{\mathrm{angle}} (\theta_i - \theta^o_i)^2, \tag{S28}$$

where θ_i is the i-th angle, and θ_i^o is the native angle value measured from the input native structure. By default $k_{\text{angle}} = 100$ kJ/mol/rad^2.

U_{dihedral} is the dihedral potential defined as

$$U_{\text{dihedral}} = \sum_i \sum_{n=1,3} k_{\text{dihedral},n}[1 + \cos(n(\theta_i - \theta_i^o - \pi))] \tag{S29}$$

where θ_i is the i-th dihedral, and θ_i^o is the dihedral measured from the input native structure. $k_{\text{dihedral},1} = 2.5$ kJ/mol, and $k_{\text{dihedral},3} = 1.25$ kJ/mol.

The memory potential U_{memory} includes Gaussian functions and stabilizes ordered domains. The native pairs are found by the shadow algorithm, and the potential is defined as

$$U_{\text{memory}} = \sum_{\substack{\text{native pairs } (i,j) \\ i<j}} -\epsilon_G \exp\left(-\frac{(r_{ij} - \mu_{ij})^2}{2\sigma_G^2}\right) + \frac{\alpha_G}{r_{ij}^{12}}\left[1 - \exp\left(-\frac{(r_{ij} - \mu_{ij})^2}{2\sigma_G^2}\right)\right], \tag{S30}$$

where $\epsilon_G = 2.5$ kJ/mol, $\alpha_G = 4.194304 \times 10^{-5}$ kJ·nm^{12}/mol, and $\sigma_G = 0.05$ nm. μ_{ij} is the distance between the i-th and j-th Cα atoms measured from the native structure. The potential has cutoff distances as $\mu_{ij} + 6\sigma_G$. We note that when modeling proteins with disordered regions, native pairs should be limited only to amino acid pairs from the folded domains.

The contact potential is modeled with the Lennard-Jones (LJ) potential as

$$U_{\text{contact}} = \sum_{j>i+3} 4\epsilon_{ij}\left[\left(\frac{\sigma}{r}\right)^{12} - \left(\frac{\sigma}{r}\right)^6\right], \tag{S31}$$

where $\sigma = 0.5$ nm and the cutoff is 1.25 nm. Values for ϵ_{ij} are defined using a scaled Miyazawa-Jernigan (MJ) statistical potential and provided in I. This potential is essential for describing the interactions between protein domains and across protein molecules.

Electrostatic interactions are described with the Debye-Hückel potential

$$U_{\text{electrostatics}} = \sum_{j>i+3} \frac{Q_i Q_j}{4\pi\epsilon_0 \epsilon r} \exp(-r/\lambda_D), \tag{S32}$$

where Q_i and Q_j are the charges of residues i and j, respectively. ϵ_0 is the vaccum permittivity and $\epsilon = 78.0$ is the relative permittivity of water. λ_D is the Debye length computed as

$$\lambda_D = \sqrt{\frac{k_B T \epsilon_0 \epsilon}{2 N_A c e^2}} \tag{S33}$$

where the variables are the Boltzmann constant (k_B), temperature (T), the Avogadro constant (N_A), the monovalent salt concentration (c), and the elementary charge (i.e. proton charge) (e). The arginine and lysine Cα atoms have $+1$ e charges, the aspartic acid and glutamic acid Cα atoms possess -1 e charges, and other amino acid Cα atoms have zero charges.

SMOG Protein-3SPN2 DNA interactions

SMOG and 3SPN2 are combined in OpenABC. Such implementations are suitable for studying protein-DNA complexes such as nucleosomes. Here we only elaborate the parameters for B-curved DNA.

The protein-DNA nonbonded contact interactions between Cα and any DNA CG atom also follow equation S31, with $\epsilon_{ij} = 0.125$ kJ/mol, $\sigma = 0.57$ nm, and cutoff equal to 1.425 nm.

The protein-DNA electrostatic interactions also follow equation S32. $+e$ charge is assigned to arginine and lysine Cα atoms, $-e$ charge is assigned to aspartic acid and glutamic acid Cα atoms, and phosphate. All the other CG atoms have zero charge.

Details for Preparing and Performing Simulations

Python implementation of the shadow algorithm for native pair identification

MOFF uses native pairs identified in the input atomistic configuration with the shadow algorithm[S11] to stabilize secondary and tertiary structures. We provide a Python implementation of the shadow algorithm to avoid the installation of Perl, which is the programming language used in the original implementation.[S12]

The shadow algorithm searches contacts between residues based on atomistic configurations. Residues α and β are in contact if any heavy atom i from residue α contacts with any heavy atom j from residue β. To test if atom i contacts with atom j, imagine there are light sources at the center of the two atoms. If neither the light from i to j nor the light from j to i is blocked by a third heavy atom k, then atoms i and j are in contact. We further provide pseudocode below to clarify the implementation of the algorithm.

Note the shadow algorithm requires atomistic models as inputs, while outputs contacts between residues. The algorithm also ignores contacts between residues that are involved in bond, angle, or dihedral potentials in the CG force field (i.e. residues that are in 1-2, 1-3, or 1-4 relations).

Algorithm 1 Pseudocode for shadow algorithm implementation. r_c is the cutoff distance for searching close neighbors. We used the MDTraj package[S13] to load and parse the initial input PDB structure, and the $O(N)$ complexity cell list spatial neighbor searching is achieved by MDAnalysis.[S14,S15] By default $r_c = 0.6$ nm, $r = 0.1$ nm, and $r_b = 0.05$ nm.

Input: $\{\vec{x}_i\}$ $(i = 1, \cdots, N)$ *# input atomistic coordinate*
Input: r_c, r, r_b *# set cutoff r_c, atom radius r, and bonded atom radius r_b*

Find all the spatially close heavy atom pairs NeighborHeavyAtomPairs $= \{(i, j)\}_{d_{ij} < r_c}$ **and** $i < j$ *# use cell list to search spatially close pairs with $O(N)$ complexity*
create empty list ResiduePairs = []
for (i, j) in NeighborHeavyAtomPairs **do**
    if not (i and j are from same residue **or** i and j are from residues with 1-2, 1-3, or 1-4 relation **or** $d_{ij} < r$ **or** (Residue(i), Residue(j)) in ResiduePairs) **then**
        # Residue(i) means the residue that atom i is from
        # ensure i and j are from different residues without bonded interactions and $d_{ij} \geq r$
        Flag = True, $r_i = r$, $r_j = r$
        Find all heavy atoms k ($k \neq i$ **and** $k \neq j$) that $d_{ik} < d_{ij}$ **and** $d_{jk} < d_{ij}$, the set including all such atoms k is called HeavyAtomNeighbors(i, j) *# find all the blocking candidates*
        for k in HeavyAtomNeighbors(i, j) **do**
            if k is connected to i **or** k is connected to j **then**
                $r_k = r_b$
            else
                $r_k = r$
            end if
            # test if any atom k blocks the contact between i and j
            if $r_k > d_{ik}$ **or** $r_k > d_{jk}$ **then**
                Flag = False
                Break
            else
                # ensure $r_k \leq d_{ik}$ and $r_k \leq d_{jk}$, which is required by LightIsBlocked
                if LightIsBlocked(i, j, k, r_j, r_k) **or** LightIsBlocked(j, i, k, r_i, r_k) **then**
                    # see the next algorithm for the definition of function LightIsBlocked
                    Flag = False
                    Break
                end if
            end if
        end for
        if flag **then**
            Add (Residue(i), Residue(j)) to ResiduePairs
        end if
    end if
end for
Output: ResiduePairs

Algorithm 2 Define function LightIsBlocked

Define LightIsBlocked(i, j, k, r_j, r_k) *# check if the light from atom i to j is blocked by atom k*
Require: $r_j \leq d_{ij}$ and $r_k \leq d_{ik}$ *# ensure* arcsin *can be computed properly*
θ_{jik} is the angle of j-i-k
if $\arcsin(r_j/d_{ij}) + \arcsin(r_k/d_{ik}) \geq \theta_{jik}$ **then**
    return True *# k blocks the light from i to j*
else
    return False
end if

Implementation of the temperature replica exchange algorithm

We implemented the temperature replica exchange[S16] simulation protocol in OpenABC. We used "torch.distributed" package to enable communications between replicas. The replica exchange is executed by exchanging coordinates and rescaled velocities while the replica temperatures are fixed. The exchange is accepted with probability determined by the Metropolis-Hastings algorithm. For example, for two replicas with indices 0 and 1, each one has temperature, coordinates, potential energy, and velocities as (T_0, x_0, E_0, v_0) and (T_1, x_1, E_1, v_1), respectively. If an exchange is attempted, the acceptance probability is $\min(1, \exp((\beta_1 - \beta_0)(E_1 - E_0)))$, where $\beta_i = 1/(k_B T_i)$ and k_B is the Boltzmann constant. If the exchange is accepted, the corresponding quantities will become $(T_0, x_1, E_1, v_1\sqrt{T_0/T_1})$ and $(T_1, x_0, E_0, v_0\sqrt{T_1/T_0})$. Rescaling the velocities ensures that the average kinetic energy is consistent with the new temperature. The Debye length is always computed at a temperature of 300 K, even if the replica thermostat has a different temperature, so the Hamiltonian of different replicas is the same.

Building and relaxing atomistic structures from coarse-grained configurations

We used "reconstruct atomic model from reduced representation (REMO)" to reconstruct atomic configurations of protein condensates.[S17] Given a coarse-grained configuration with only coordinates for α carbons, REMO first removes steric clashes between these atoms. It then reconstructs atomistic representations by optimizing the hydrogen bond networks with backbone built from a backbone isomer library. We iteratively applied REMO to individual chains of the condensate system for improved computation efficiency (see Algorithm 3).

To perform atomistic simulations starting from the reconstructed structure, we solvated it with explicit water molecules and counter ions. The box size was chosen such that protein atoms are at least 2 nm from the boundary, resulting in a size of 39.887 × 44.420 × 45.281

nm^3. The concentration of monovalent ions was set as 82 mM NaCl.

From the solvated structure, we first carried out an energy minimization using a double-precision version of GROMACS. Subsequently, using mixed-precision GROMACS, we performed a 62.5-ps-long NVT simulation with a timestep of 0.5 fs, followed by a 1-ns-long NVT simulation with a timestep of 1 fs. Finally, we performed the production simulation for more than 20 ns with a timestep of 1.5 fs. The temperature was maintained at 260 K during simulations using the velocity rescaling[S18] scheme with a coupling constant of 1 ps. 240 CPU cores were used to perform the atomistic simulation.

Algorithm 3 Reconstruct full atomic configurations for proteins

Input: $CGCoords = \{\vec{x}_i\}$ $(i = 1, \cdots, N)$ *# input Cα coordinates of the system*
Input: n *# number of protein chains*
Input: $NumRes = \{N_j\}(\sum_j^n N_j = N)$ *# number of residues in each chain*

Create empty atomic coordinates list $AtomicCoords = []$
if $n == 1$ **then**
  $AtomicCoords = \mathrm{REMO}(CGCoords)$ *# there is only one chain, so reconstruct using REMO model directly*
else if $n > 1$ **then**
  $CGCoordsList = split(CGCoords, NumRes)$ *# there are multiple protein chains, so split the Cα coordinates of the whole system into n coordinate lists $CGCoordsList = [CGCoords\_1, ..., CGCoords\_n]$.*
  for $CGCoords\_i$ in $CGCoordsList$ **do**
    $AtomicCoords\_i = REMO(CGCoords\_i)$ *# reconstruct i-th protein chain using REMO*
    $AtomicCoords\_i = align(AtomicCoords\_i, CGCoords\_i)$ *# align atomic coordinates of i-th chain with its CG cooridnates*
  end for
  $AtomicCoords = combine([AtomicCoords\_1, ..., AtomicCoords\_n])$ *# combine atomic coordinates of all the protein chains together*
end if

Output: $AtomicCoords$

Setting up MOFF HP1 system

Setting up MD simulations with MOFF requires 3D protein native configurations as inputs. We followed the protocols established before[S1,S8] to build HP1 dimer structures. We first

built the monomeric atomistic structures with RaptorX.[S19] Each monomer contains two ordered domains: chromodomain (CD) and chromoshadow domain (CSD). The CD and CSD of HP1α correspond to regions with residues 17-72 and residues 115-176, respectively, while the CD and CSD of HP1β correspond to regions with residues 21-79 and residues 117-175. To build dimer structures, we aligned the CSDs of two monomers to the bound configuration reported in PDB ID 3I3C for HP1α and PDB ID 3Q6S for HP1β.

From the constructed atomistic structures for HP1 dimers, we determined the equilibrium angles, dihedrals, and native pair distances with MDTraj[S13] to define the corresponding terms in the force field. We only included native pairs between residues from the same CD, the same CSD, or between two CSDs. For HP1β, the initial dimer structure includes unphysical overlaps between the C-terminal tail and CSD from different monomers. To avoid the impact of these overlaps on native pair detection, we applied the shadow algorithm to a single monomer to determine intra-CD and CSD native pairs. For native pairs at the dimer interface between two CSDs, we applied the shadow algorithm to a structure containing only the two CSDs.

The interaction strength in the native pair potential was set as $\epsilon = 6$ kJ/mol (equation S5). We used a concentration of 82 mM monovalent salt to compute the Debye length (equation S9) and to closely mimic the experimental setup.[S20]

Benchmarking the performance of condensate simulations

We carried out simulations of condensate systems with N_1 HP1α dimers and N_2 200-bp-long dsDNA using both GROMACS and OpenMM. GROMACS version is 2018.4 and compiled with mixed precision. The precision style for OpenMM was set as "mixed", which means single precision for forces while double precision for integration.

For CPU simulations with GROMACS, we used the leap-frog stochastic dynamics integrator (sd integrator) with the coupling constant (tau-t) set as 1 ps. We used the repository that implements the MOFF and MRG-CG DNA force field available at `https://github.`

com/ZhangGroup-MITChemistry/MOFF. For single-GPU simulations with OpenMM, the integrator was set as Langevin middle integrator with a friction coefficient of 1 ps^{-1}. All simulations were performed under temperature 300 K and lasted one million steps with a timestep of 10 fs.

Validating the force field implementation in OpenMM

To validate our implementation MOFF, we ran a 0.1-million-step NVT simulation for HP1α dimer with GROMACS at 300 K to collect ten configurations. We used sd integrator with time coupling constant as 1 ps and a timestep of 10 fs. We then evaluated the potential energies for the ten configurations using both OpenMM and GROMACS with a salt concentration of 82 mM and temperature at 300 K. The interaction strength for protein native pair contacts was set as $\epsilon = 6.0$ kJ/mol. The results are shown in J, and the energies computed from the two software agree well. The minor differences come from using tabulated functions for native pair, contact, and electrostatic potentials in GROMACS but analytical expressions in OpenMM. In addition, GROMACS does not shift nonbonded contact potentials to zero at cutoff distances, while OpenMM does.

To validate the MRG implementation and its integration with MOFF, we simulated a protein-DNA complex (HP1α dimer + 200-bp-long dsDNA) to collect ten configurations. An umbrella bias on the center of mass (COM) distance, r_{COM} defined as

$$U_{\mathrm{bias}} = \frac{k_{\mathrm{bias}}}{2} r_{\mathrm{COM}}^2 \tag{S34}$$

was applied to promote protein-DNA contacts, with $k_{\mathrm{bias}} = 50$ kJ/mol/nm^2. The simulation was performed with GROMACS at 300 K and lasted 2 million steps, with configurations saved at every 0.2 million steps. The timestep is 10 fs and the time coupling constant is 1 ps. The umbrella bias was implemented using PLUMED.[S21] We then evaluated the potential energies for the ten configurations using both OpenMM and GROMACS with a

salt concentration of 82 mM and temperature at 300 K. The interaction strength for protein native pair contacts was set as $\epsilon = 6.0$ kJ/mol. The results are shown in K and agree well. Again, the use of tabulated functions in GROMACS resulted in minor differences.

To verify our implementation of the HPS force field, we ran a 0.1-million-step simulation for DDX4 with OpenMM to collect ten configurations. We used Langevin middle integrator with friction coefficient as 1/ps and a timestep of 10 fs, with configurations saved at every 10,000 steps. We evaluated the potential energies for the ten configurations using both OpenMM and HOOMD-Blue. We tested both Urry and KR scales with the optimal parameter set ($\mu_{\mathrm{Urry}}^{\mathrm{opt}} = 1$, $\Delta_{\mathrm{Urry}}^{\mathrm{opt}} = 0.08$ and $\mu_{\mathrm{KR}}^{\mathrm{opt}} = 1$, $\Delta_{\mathrm{KR}}^{\mathrm{opt}} = 0$). Since HOOMD-Blue does not shift the potential to ensure continuity at cutoff distances, we did not offset nonbonded potentials in OpenMM for energy comparisons. The energies computed with OpenMM and HOOMD-Blue match exactly as shown in L.

To validate our implementation of the Mpipi force field, we ran a 1-million-step-long simulation for a polyR+polyK+polyU system with LAMMPS. The system consists of a chain of 10 arginines, a chain of 10 lysines, and 2 individual chains of 10 uracils. The simulation was performed with Langevin dynamics at 300 K, with a damping coefficient of 10.001 ps and a timestep of 10 fs. The configurations were saved every 0.1 million steps. We evaluated the interaction energies for the 10 configurations using both OpenMM and LAMMPS and the results as shown in M.

Computing DNA persistence length with the MRG-CG model

To compute the DNA persistence length, we simulated a 200-bp dsDNA chain with the MRG-CG model at 300 K with a monovalent salt concentration of 100 mM. The dsDNA was placed into a cubic box of length 500 nm. We performed three independent 5-billion-step NVT simulations using the Langevin middle integrator with a friction coefficient of 1/ps and a timestep of 10 fs. The configurations were saved every 0.1 million steps, and the first one billion steps were discarded as equilibration.

Using the simulated configurations, we computed the persistence length as follows. Since DNA is a double-strand helix with a periodicity of about 10 bp, we choose CG particles of index 66, 76, 86, 96, 106, 116, 126, 136 to define a pseudo chain. These particles are within the middle 70 bp of the DNA, thus avoiding boundary effects. By connecting neighboring particles on the pseudo chains, we can define a bond vector $\vec{b}_i$ as the normalized vector of pointing from the $i-1$-th to the i-th particle. The average correlation between two normalized pseudo bond vectors with gap n was computed as

$$C(n) = \left\langle \vec{b}_i \cdot \vec{b}_{i+n} \right\rangle \tag{S35}$$

where the average was performed over pseudo bond index i and all the configurations. Assuming an exponential decay for $C(n) \approx \exp\left(-n\bar{l}_b/l_p\right)$, we determined the persistence length with numerical fitting of $\log C(n)$ and n as

$$\log C(n) = -\alpha n + \beta. \tag{S36}$$

$\alpha = \bar{l}_b/l_p$ with $\bar{l}_b$ as the mean bond length along the pseudo chain. The mean bond length $\bar{l}_b$ was 3.37 nm, and the persistence length l_p was 48.83 ± 2.71 nm. The fitting results are shown in Figure A.

Sequences

The following sequences were used in simulations of respective proteins.

HP1α monomer

MGKKTKRTADSSSSEDEEEYVVEKVLDRRVVKGQVEYLLKWKGFSEEHNTWEPEKN
LDCPELISEFMKKYKKMKEGENNKPREKSESNKRKSNFSNSADDIKSKKKREQSND
IARGFERGLEPEKIIGATDSCGDLMFLMKWKDTDEADLVLAKEANVKCPQIVIAFY

EERLTWHAYPEDAENKEKETAKS

HP1β monomer

MGKKQNKKKVEEVLEEEEEEYVVEKVLDRRVVKGKVEYLLKWKGFSDEDNTWEPEE
NLDCPDLIAEFLQSQKTAHETDKSEGGKRKADSDSEDKGEESKPKKKKEESEKPRG
FARGLEPERIIGATDSSGELMFLMKWKNSDEADLVPAKEANVKCPQVVISFYEERL
TWHSYPSEDDDKKDDKN

FUS LC

MASNDYTQQATQSYGAYPTQPGQGYSQQSSQPYGQQSYSGYSQSTDTSGYGQSSYS
SYGQSQNTGYGTQSTPQGYGSTGGYGSSQSSQSSYGQQSSYPGYGQQPAPSSTSGS
YGSSSQSSSYGQPQSGSYSQQPSYGGQQQSYGQQQSYNPPQGYGQQNQYNS

DDX4

MGDEDWEAEINPHMSSYVPIFEKDRYSGENGDNFNRTPASSSEMDDGPSRRDHFMK
SGFASGRNFGNRDAGECNKRDNTSTMGGFGVGKSFGNRGFSNSRFEDGDSSGFWRE
SSNDCEDNPTRNRGFSKRGGYRDGNNSEASGPYRRGGRGSFRGCRGGFGLGSPNND
LDPDECMQRTGGLFGSRRPVLSGTGNGDTSQSRSGSGSERGGYKGLNEEVITGSGK
NSWKSEAEGGES

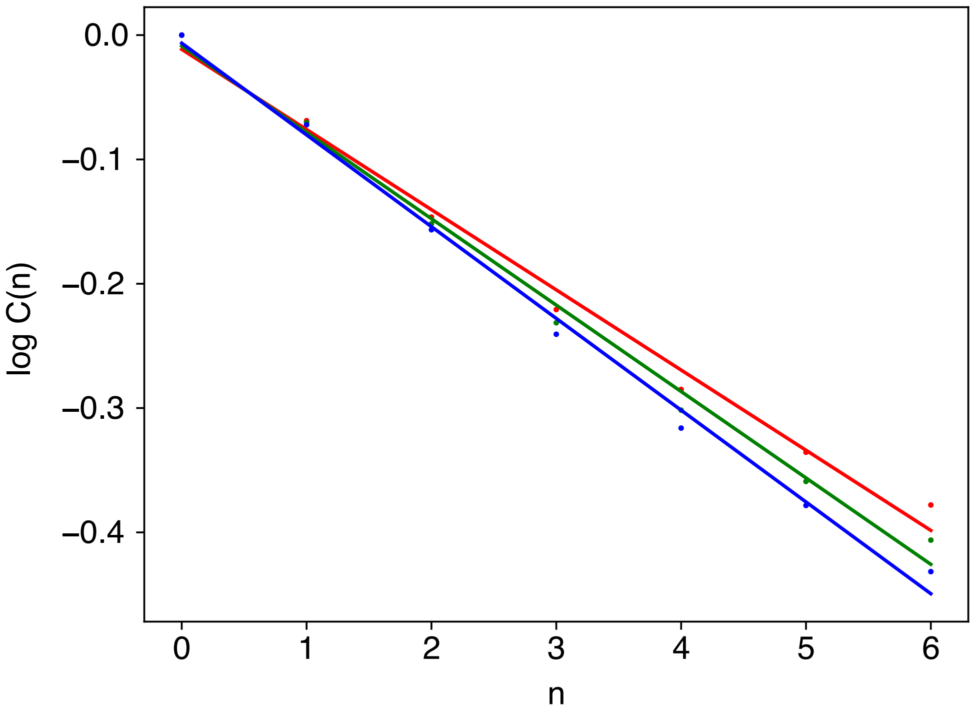

Fig A: The log of the bond vector correlation, $\log C(n)$, as a function of the bond separation n. The dots were obtained from MD simulations, with three colors indicate three independent simulations. The lines are numerical fits to the data. See text *Section: Computing DNA persistence length with the MRG-CG model* for simulation details and computing persistence length from the numerical fitting.

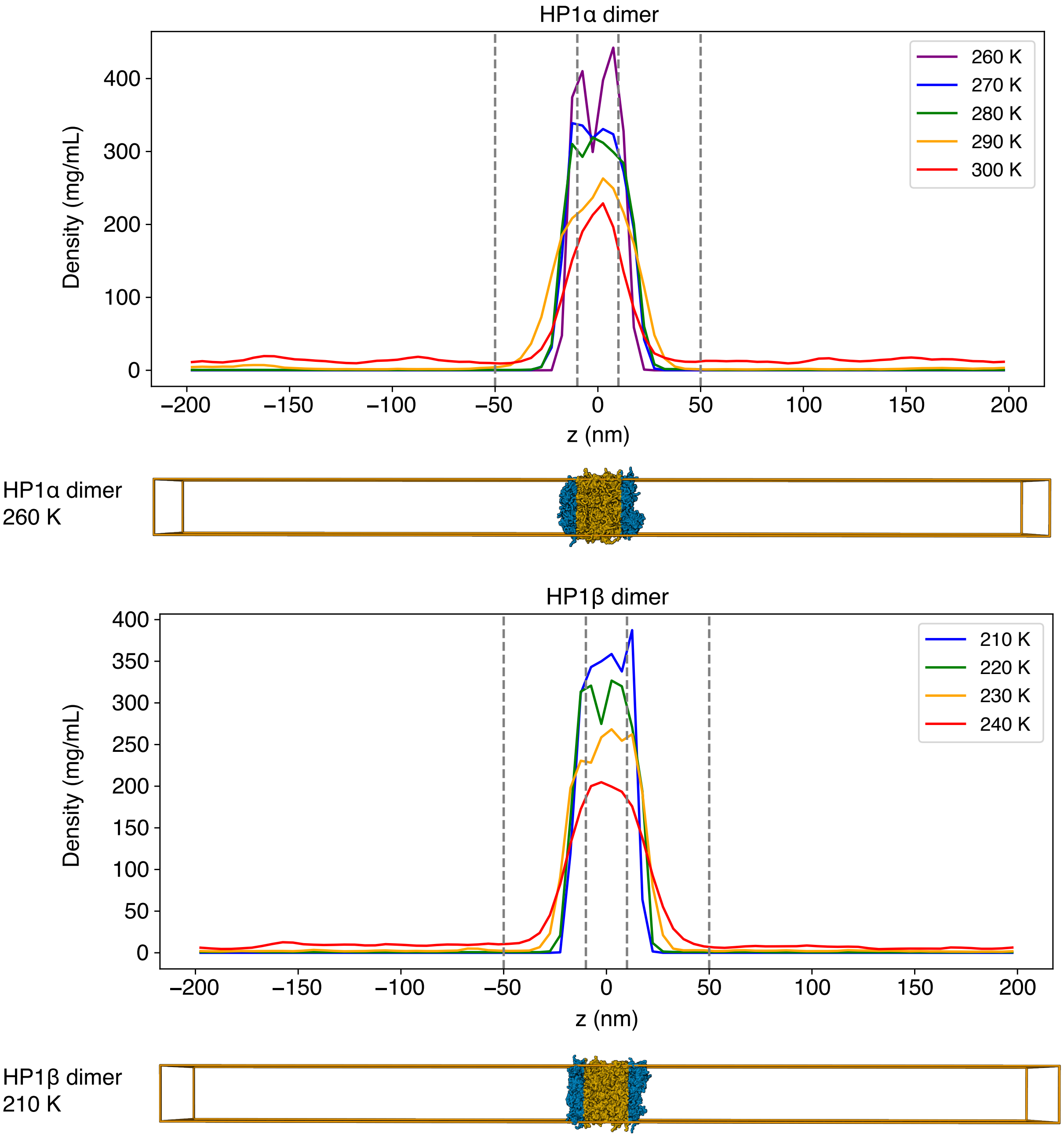

Fig B: Density profiles obtained from slab simulations of HP1α (left) and HP1β (right) dimers with the MOFF model. Vertical lines are set at $z = \pm 10$ and ± 50 nm. The final snapshots of the slab simulations at 260 K for HP1α and 210 K for HP1β are shown. CG atoms with $|z| < 10$ nm are colored in yellow, while the remaining are shown in blue.

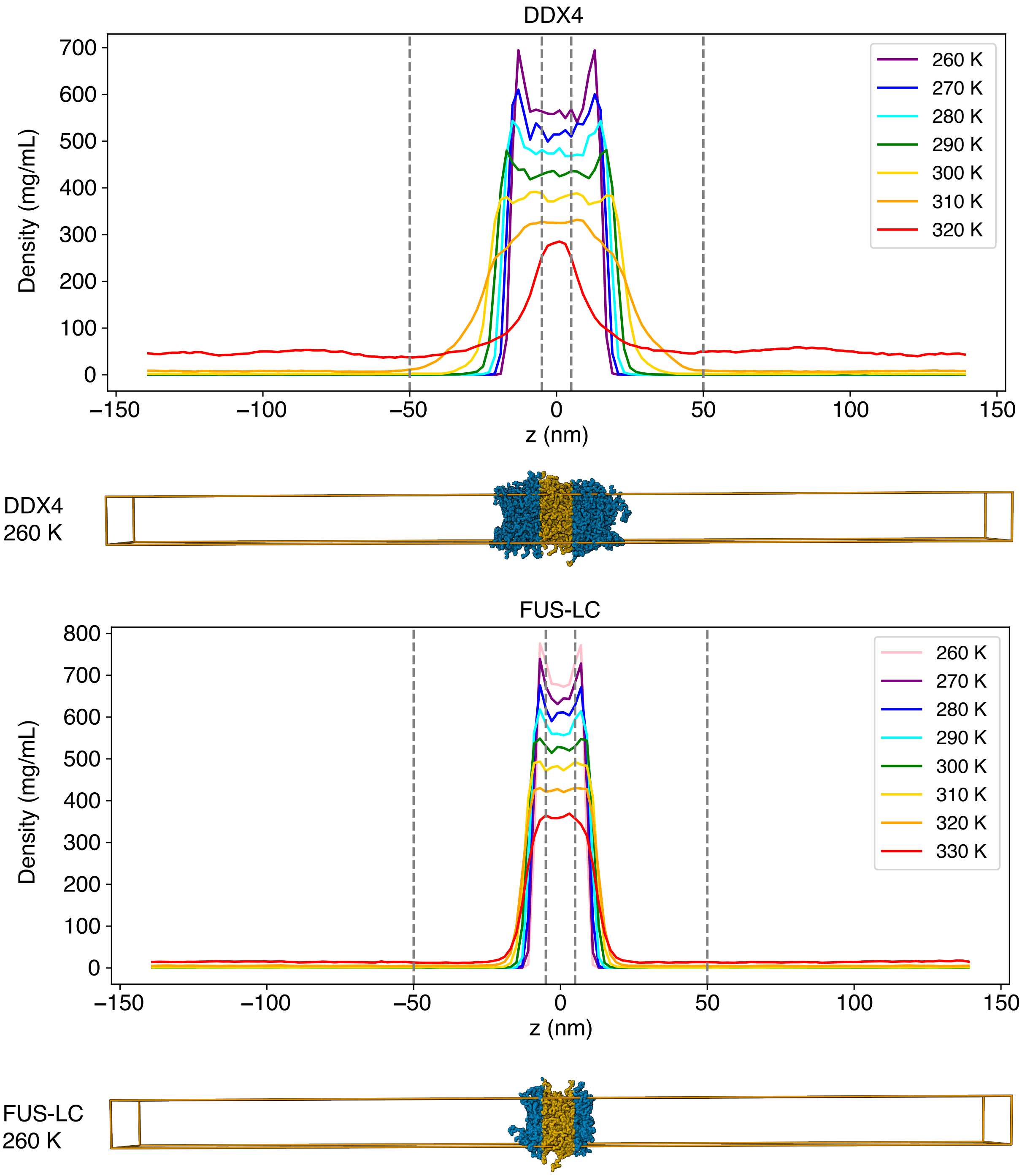

Fig C: Density profiles obtained from slab simulations of DDX4 and FUS LC with the HPS model using the Urry scale optimal parameter set ($\mu = \mu_{\mathrm{Urry}}^{\mathrm{opt}} = 1$ and $\Delta = \Delta_{\mathrm{Urry}}^{\mathrm{opt}} = 0.08$) at different temperatures. Vertical dashed lines are set at $z = \pm 5$ nm and ± 50 nm. The final snapshots of the slab simulations at 260 K are shown. CG atoms with $|z| < 5$ nm are colored in yellow, while the remaining are shown in blue. This figure shows that the $|z| < 5$ nm and $|z| > 50$ nm regimes can represent the concentrated and dilute phases, respectively.

Table A: The amino acid mass, sizes, and charges used by MOFF and HPS models. Both models share the same amino acid mass and sizes. The charge of HIS differs in the two models, while other amino acids share the same charge. Here e is the elementary charge.

Amino acid	Mass (Da)	Size (nm)	MOFF charge (e)	HPS charge (e)
ALA	71.08	0.504	0	0
ARG	156.20	0.656	1	1
ASN	114.10	0.568	0	0
ASP	115.10	0.558	-1	-1
CYS	103.10	0.548	0	0
GLN	128.10	0.602	0	0
GLU	129.10	0.592	-1	-1
GLY	57.05	0.450	0	0
HIS	137.10	0.608	0.25	0.5
ILE	113.20	0.618	0	0
LEU	113.20	0.618	0	0
LYS	128.20	0.636	1	1
MET	131.20	0.618	0	0
PHE	147.20	0.636	0	0
PRO	97.12	0.556	0	0
SER	87.08	0.518	0	0
THR	101.10	0.562	0	0
TRP	163.20	0.678	0	0
TYR	163.20	0.646	0	0
VAL	99.70	0.586	0	0

Table B: MOFF protein contact ϵ_{ij} values as defined in equation S6. Due to limited space, the numbers are rounded to 3 decimal places. The values are in unit kJ/mol.

	ALA	ARG	ASN	ASP	CYS	GLN	GLU	GLY	HIS	ILE	LEU	LYS	MET	PHE	PRO	SER	THR	TRP	TYR	VAL
ALA	0.904	0.022	1.413	-0.436	1.376	0.025	-0.243	-0.439	-0.023	2.340	-0.111	-0.201	1.209	1.805	-0.178	-0.136	-0.210	3.887	3.467	1.320
ARG	-	5.746	-2.640	-0.566	0.295	1.583	-0.919	2.450	2.005	4.739	1.868	-1.751	-0.551	-0.432	-1.081	-1.697	-0.411	2.862	6.503	3.243
ASN	-	-	-0.429	1.105	4.326	0.400	-1.681	-0.168	-0.507	-0.763	-0.293	1.086	-1.218	-0.199	-2.204	-0.311	-0.156	3.380	-0.918	-0.383
ASP	-	-	-	0.961	1.460	-0.381	1.739	-1.073	0.396	-0.094	-0.398	-0.993	-1.507	-0.514	-0.802	-0.257	2.585	-0.523	-0.911	-0.158
CYS	-	-	-	-	5.406	-1.510	3.079	0.322	2.041	-0.628	3.885	2.836	3.098	0.518	1.456	0.965	5.066	0.512	6.124	2.614
GLN	-	-	-	-	-	-0.720	-1.840	0.111	-0.197	3.443	1.313	2.394	6.734	-0.405	-1.431	-0.628	1.014	-0.926	4.234	1.435
GLU	-	-	-	-	-	-	-1.763	0.041	-2.409	2.840	-0.210	-0.956	4.629	-0.544	-0.765	-0.195	1.636	2.347	1.963	-0.278
GLY	-	-	-	-	-	-	-	1.059	-0.009	0.603	0.338	0.549	-2.221	-0.269	-0.128	-0.832	0.853	2.307	-0.653	0.386
HIS	-	-	-	-	-	-	-	-	-0.068	1.993	-0.251	4.281	-0.595	2.827	2.334	0.438	0.855	-0.349	-0.281	2.910
ILE	-	-	-	-	-	-	-	-	-	3.144	5.030	1.627	3.709	2.266	1.182	2.246	3.081	-0.778	6.530	3.399
LEU	-	-	-	-	-	-	-	-	-	-	-0.100	1.173	5.956	5.702	-0.187	-0.172	-0.163	6.058	-0.659	0.649
LYS	-	-	-	-	-	-	-	-	-	-	-	0.037	-0.366	-0.048	-0.401	0.286	-0.841	8.442	-0.037	0.652
MET	-	-	-	-	-	-	-	-	-	-	-	-	-0.305	1.530	-1.966	-0.350	2.956	4.745	-0.017	5.035
PHE	-	-	-	-	-	-	-	-	-	-	-	-	-	2.684	2.241	1.147	3.540	7.383	6.564	2.303
PRO	-	-	-	-	-	-	-	-	-	-	-	-	-	-	-1.197	-0.524	-0.477	0.185	0.859	-1.460
SER	-	-	-	-	-	-	-	-	-	-	-	-	-	-	-	0.337	1.024	4.761	1.575	0.241
THR	-	-	-	-	-	-	-	-	-	-	-	-	-	-	-	-	0.764	2.486	2.206	-0.134
TRP	-	-	-	-	-	-	-	-	-	-	-	-	-	-	-	-	-	5.889	7.143	3.585
TYR	-	-	-	-	-	-	-	-	-	-	-	-	-	-	-	-	-	-	5.901	0.489
VAL	-	-	-	-	-	-	-	-	-	-	-	-	-	-	-	-	-	-	-	2.327

Table C: MRG DNA model bond parameters. All the $k_{\text{bond},n}$ are in unit kcal/mol/nm^2, and r_0 unit is nm.

$k_{\text{bond},2}$	$k_{\text{bond},3}$	$k_{\text{bond},4}$	r_0
262.5	-226	149	0.496

Table D: MRG DNA model angle parameters. All the $k_{\text{angle},n}$ are in unit kcal/mol/degree^2, and θ_0 unit is degree.

$k_{\text{angle},2}$	$k_{\text{angle},3}$	$k_{\text{angle},4}$	θ_0
9.22	4.16	1.078	156

Table E: MRG DNA model fan bond parameters. All the $k_{\text{fan bond},n}$ are in kcal/mol/nm^2, and $r_{\Delta,0}$ unit is nm. Δ means the fan bond between CG nucleotide i and $j + \Delta$, where nucleotide i and j are WC-paired.

Δ	$k_{\text{bond},2}$	$k_{\text{bond},3}$	$k_{\text{bond},4}$	$r_{\Delta,0}$
-5	4.67	2.1	1.46	1.71
-4	0.0001324	-12.2	18.5	1.635
-3	8.5	-44.4	50.0	1.47
-2	12.3	-40.0	37.0	1.345
-1	4.0	-10.0	8.0	1.23
0	292.0	410.0	720.0	1.13
1	11.5	-41.0	58.0	0.99
2	9.55	-45.9	50.2	0.92
3	13.78	-52.7	50.0	1.02
4	13.86	-56.8	50.0	1.25
5	36.26	-77.0	50.0	1.69

Table F: The normalized KR scale and Urry hydropathy scale values (i.e. λ_i parameters in equation S14).

Amino acid	KR scale	Urry scale
ALA	0.730	0.602942
ARG	0.0	0.558824
ASN	0.432	0.588236
ASP	0.378	0.294119
CYS	0.595	0.64706
GLN	0.514	0.558824
GLU	0.459	0.0
GLY	0.649	0.57353
HIS	0.514	0.764707
ILE	0.973	0.705883
LEU	0.973	0.720589
LYS	0.514	0.382354
MET	0.838	0.676471
PHE	1.0	0.82353
PRO	1.0	0.758824
SER	0.595	0.588236
THR	0.676	0.588236
TRP	0.946	1.0
TYR	0.865	0.897059
VAL	0.892	0.664707

Table G: Mpipi parameter ϵ values as defined in equation S25. Due to limited space, the numbers are rounded to 3 decimal places. The values are in unit kJ/mol.

	ALA	ARG	ASN	ASP	CYS	GLN	GLU	GLY	HIS	ILE	LEU	LYS	MET	PHE	PRO	SER	THR	TRP	TYR	VAL	A	C	G	U
ALA	0.207	0.738	0.509	0.327	0.249	0.523	0.345	0.305	0.872	0.104	0.127	0.158	0.186	0.923	0.268	0.232	0.168	1.255	0.980	0.115	1.175	0.804	1.183	0.742
ARG	-	0.376	1.040	0.006	0.780	1.054	0.007	0.837	0.520	0.636	0.658	0.510	0.718	2.280	0.799	0.764	0.699	2.894	2.543	0.647	2.518	1.778	2.535	1.652
ASN	-	-	0.811	0.629	0.551	0.825	0.647	0.607	1.174	0.406	0.429	0.460	0.488	1.225	0.570	0.534	0.470	1.557	1.282	0.417	1.477	1.106	1.485	1.044
ASP	-	-	-	0.331	0.368	0.643	0.345	0.425	0.007	0.224	0.246	0.002	0.306	1.043	0.388	0.352	0.288	1.375	1.100	0.235	1.237	0.866	1.245	0.804
CYS	-	-	-	-	0.290	0.564	0.387	0.347	0.914	0.146	0.168	0.199	0.228	0.964	0.310	0.274	0.209	1.296	1.022	0.157	1.216	0.846	1.224	0.783
GLN	-	-	-	-	-	0.839	0.661	0.621	1.188	0.420	0.442	0.473	0.502	1.239	0.584	0.548	0.484	1.571	1.296	0.431	1.490	1.120	1.499	1.057
GLU	-	-	-	-	-	-	0.358	0.444	0.007	0.243	0.265	0.002	0.325	1.061	0.406	0.371	0.306	1.393	1.119	0.253	1.250	0.880	1.259	0.817
GLY	-	-	-	-	-	-	-	0.404	0.970	0.203	0.225	0.256	0.285	1.021	0.366	0.331	0.266	1.353	1.079	0.213	1.273	0.903	1.281	0.840
HIS	-	-	-	-	-	-	-	-	0.114	0.769	0.792	0.366	0.851	1.588	0.933	0.898	0.833	1.920	1.646	0.780	1.128	0.758	1.136	0.695
ILE	-	-	-	-	-	-	-	-	-	0.002	0.024	0.055	0.084	0.820	0.165	0.130	0.065	1.152	0.878	0.012	1.072	0.702	1.080	0.639
LEU	-	-	-	-	-	-	-	-	-	-	0.046	0.077	0.106	0.842	0.188	0.152	0.087	1.174	0.900	0.035	1.094	0.724	1.102	0.661
LYS	-	-	-	-	-	-	-	-	-	-	-	0.080	0.137	0.482	0.219	0.183	0.118	0.438	0.430	0.066	0.667	0.444	0.672	0.407
MET	-	-	-	-	-	-	-	-	-	-	-	-	0.166	0.902	0.247	0.212	0.147	1.234	0.960	0.094	1.154	0.784	1.162	0.721
PHE	-	-	-	-	-	-	-	-	-	-	-	-	-	1.639	0.984	0.948	0.884	1.971	1.696	0.831	1.890	1.520	1.899	1.457
PRO	-	-	-	-	-	-	-	-	-	-	-	-	-	-	0.329	0.293	0.229	1.316	1.042	0.176	1.236	0.865	1.244	0.803
SER	-	-	-	-	-	-	-	-	-	-	-	-	-	-	-	0.258	0.193	1.280	1.006	0.141	1.200	0.830	1.208	0.767
THR	-	-	-	-	-	-	-	-	-	-	-	-	-	-	-	-	0.129	1.216	0.941	0.076	1.135	0.765	1.144	0.702
TRP	-	-	-	-	-	-	-	-	-	-	-	-	-	-	-	-	-	2.302	2.028	1.163	2.222	1.852	2.231	1.789
TYR	-	-	-	-	-	-	-	-	-	-	-	-	-	-	-	-	-	-	1.754	0.889	1.948	1.578	1.956	1.515
VAL	-	-	-	-	-	-	-	-	-	-	-	-	-	-	-	-	-	-	-	0.023	1.083	0.712	1.091	0.650
A	-	-	-	-	-	-	-	-	-	-	-	-	-	-	-	-	-	-	-	-	2.142	1.772	2.151	1.709
C	-	-	-	-	-	-	-	-	-	-	-	-	-	-	-	-	-	-	-	-	-	1.402	1.780	1.339
G	-	-	-	-	-	-	-	-	-	-	-	-	-	-	-	-	-	-	-	-	-	-	2.159	1.718
U	-	-	-	-	-	-	-	-	-	-	-	-	-	-	-	-	-	-	-	-	-	-	-	1.276

Table H: Mpipi parameter σ values as defined in equation S25. Due to limited space, the numbers are rounded to 3 decimal places. The values are in unit nm.

	ALA	ARG	ASN	ASP	CYS	GLN	GLU	GLY	HIS	ILE	LEU	LYS	MET	PHE	PRO	SER	THR	TRP	TYR	VAL	A	C	G	U
ALA	0.527	0.605	0.559	0.555	0.550	0.577	0.572	0.498	0.580	0.589	0.588	0.597	0.587	0.594	0.554	0.534	0.558	0.616	0.600	0.571	0.686	0.675	0.689	0.672
ARG	-	0.684	0.638	0.633	0.628	0.656	0.651	0.577	0.659	0.664	0.664	0.674	0.664	0.673	0.632	0.612	0.635	0.695	0.679	0.648	0.764	0.753	0.767	0.750
ASN	-	-	0.592	0.587	0.582	0.610	0.605	0.531	0.613	0.619	0.619	0.628	0.619	0.628	0.586	0.567	0.590	0.649	0.633	0.602	0.718	0.707	0.722	0.705
ASP	-	-	-	0.582	0.577	0.605	0.600	0.526	0.608	0.615	0.615	0.624	0.614	0.622	0.581	0.562	0.585	0.644	0.628	0.598	0.713	0.702	0.717	0.700
CYS	-	-	-	-	0.572	0.600	0.595	0.521	0.603	0.611	0.610	0.619	0.609	0.617	0.577	0.557	0.580	0.639	0.622	0.594	0.708	0.697	0.712	0.695
GLN	-	-	-	-	-	0.628	0.623	0.549	0.631	0.637	0.637	0.646	0.637	0.645	0.604	0.584	0.607	0.667	0.651	0.620	0.736	0.725	0.739	0.722
GLU	-	-	-	-	-	-	0.618	0.544	0.626	0.632	0.632	0.641	0.632	0.640	0.599	0.579	0.603	0.662	0.645	0.616	0.731	0.720	0.734	0.717
GLY	-	-	-	-	-	-	-	0.470	0.552	0.558	0.558	0.567	0.558	0.566	0.525	0.505	0.528	0.588	0.571	0.541	0.657	0.646	0.660	0.643
HIS	-	-	-	-	-	-	-	-	0.634	0.639	0.639	0.649	0.639	0.648	0.607	0.587	0.610	0.670	0.654	0.623	0.739	0.728	0.742	0.725
ILE	-	-	-	-	-	-	-	-	-	0.692	0.661	0.662	0.650	0.654	0.614	0.595	0.621	0.676	0.659	0.672	0.768	0.757	0.772	0.755
LEU	-	-	-	-	-	-	-	-	-	-	0.653	0.659	0.648	0.654	0.614	0.595	0.620	0.676	0.659	0.639	0.749	0.738	0.752	0.735
LYS	-	-	-	-	-	-	-	-	-	-	-	0.667	0.657	0.663	0.623	0.604	0.628	0.685	0.668	0.643	0.756	0.745	0.759	0.742
MET	-	-	-	-	-	-	-	-	-	-	-	-	0.647	0.654	0.613	0.594	0.618	0.676	0.659	0.632	0.745	0.734	0.749	0.732
PHE	-	-	-	-	-	-	-	-	-	-	-	-	-	0.663	0.621	0.602	0.625	0.685	0.668	0.637	0.753	0.742	0.757	0.740
PRO	-	-	-	-	-	-	-	-	-	-	-	-	-	-	0.581	0.561	0.584	0.643	0.627	0.597	0.712	0.701	0.716	0.699
SER	-	-	-	-	-	-	-	-	-	-	-	-	-	-	-	0.541	0.565	0.623	0.607	0.578	0.693	0.682	0.696	0.679
THR	-	-	-	-	-	-	-	-	-	-	-	-	-	-	-	-	0.589	0.646	0.630	0.604	0.716	0.705	0.720	0.703
TRP	-	-	-	-	-	-	-	-	-	-	-	-	-	-	-	-	-	0.707	0.690	0.659	0.775	0.764	0.779	0.762
TYR	-	-	-	-	-	-	-	-	-	-	-	-	-	-	-	-	-	-	0.673	0.642	0.759	0.748	0.762	0.745
VAL	-	-	-	-	-	-	-	-	-	-	-	-	-	-	-	-	-	-	-	0.627	0.735	0.724	0.739	0.722
A	-	-	-	-	-	-	-	-	-	-	-	-	-	-	-	-	-	-	-	-	0.844	0.833	0.848	0.831
C	-	-	-	-	-	-	-	-	-	-	-	-	-	-	-	-	-	-	-	-	-	0.822	0.837	0.820
G	-	-	-	-	-	-	-	-	-	-	-	-	-	-	-	-	-	-	-	-	-	-	0.851	0.834
U	-	-	-	-	-	-	-	-	-	-	-	-	-	-	-	-	-	-	-	-	-	-	-	0.817

Table I: SMOG MJ potential parameter ϵ as defined in equation S31. Due to the limited space, the numbers are rounded to 3 decimal places. The values are in unit kJ/mol.

	ALA	ARG	ASN	ASP	CYS	GLN	GLU	GLY	HIS	ILE	LEU	LYS	MET	PHE	PRO	SER	THR	TRP	TYR	VAL
ALA	0.436	0.293	0.295	0.273	0.572	0.303	0.242	0.370	0.386	0.734	0.787	0.210	0.632	0.771	0.326	0.322	0.372	0.613	0.539	0.648
ARG	-	0.249	0.263	0.367	0.412	0.289	0.364	0.276	0.346	0.582	0.646	0.095	0.500	0.638	0.273	0.260	0.305	0.547	0.507	0.492
ASN	-	-	0.269	0.269	0.415	0.274	0.242	0.279	0.334	0.520	0.600	0.194	0.473	0.601	0.245	0.253	0.301	0.492	0.443	0.454
ASP	-	-	-	0.194	0.386	0.234	0.164	0.255	0.372	0.508	0.545	0.269	0.412	0.558	0.213	0.261	0.289	0.455	0.443	0.398
CYS	-	-	-	-	0.872	0.457	0.364	0.507	0.577	0.882	0.935	0.313	0.800	0.930	0.492	0.459	0.499	0.794	0.667	0.795
GLN	-	-	-	-	-	0.247	0.228	0.266	0.318	0.589	0.648	0.207	0.529	0.657	0.277	0.239	0.305	0.499	0.476	0.492
GLU	-	-	-	-	-	-	0.146	0.196	0.345	0.524	0.576	0.289	0.463	0.571	0.202	0.237	0.279	0.479	0.447	0.428
GLY	-	-	-	-	-	-	-	0.359	0.345	0.606	0.667	0.184	0.544	0.662	0.300	0.292	0.334	0.548	0.483	0.542
HIS	-	-	-	-	-	-	-	-	0.489	0.664	0.728	0.216	0.638	0.765	0.361	0.338	0.388	0.638	0.564	0.574
ILE	-	-	-	-	-	-	-	-	-	1.049	1.129	0.483	0.965	1.097	0.603	0.564	0.646	0.927	0.842	0.970
LEU	-	-	-	-	-	-	-	-	-	-	1.182	0.540	1.028	1.167	0.674	0.629	0.696	0.985	0.909	1.039
LYS	-	-	-	-	-	-	-	-	-	-	-	0.019	0.398	0.539	0.156	0.168	0.210	0.431	0.417	0.399
MET	-	-	-	-	-	-	-	-	-	-	-	-	0.876	1.052	0.553	0.486	0.563	0.890	0.787	0.853
PHE	-	-	-	-	-	-	-	-	-	-	-	-	-	1.164	0.682	0.645	0.686	0.988	0.908	1.009
PRO	-	-	-	-	-	-	-	-	-	-	-	-	-	-	0.281	0.252	0.305	0.598	0.512	0.532
SER	-	-	-	-	-	-	-	-	-	-	-	-	-	-	-	0.268	0.314	0.479	0.446	0.489
THR	-	-	-	-	-	-	-	-	-	-	-	-	-	-	-	-	0.340	0.516	0.483	0.555
TRP	-	-	-	-	-	-	-	-	-	-	-	-	-	-	-	-	-	0.811	0.747	0.831
TYR	-	-	-	-	-	-	-	-	-	-	-	-	-	-	-	-	-	-	0.669	0.741
VAL	-	-	-	-	-	-	-	-	-	-	-	-	-	-	-	-	-	-	-	0.885

Table J: Comparison of potential energies computed with OpenMM and GROMACS using MOFF for ten configurations of HP1α dimer. The energy unit is kJ/mol. See text *Section:Validating the force field implementation in OpenMM* for simulation details.

Frame ID	Software	Bonds	Angles	Dihedrals	Native pairs	Contacts	Electrostatics
1	OpenMM	4.97	2.55	1.67	-3346.36	-103.74	-53.22
	GROMACS	4.97	2.55	1.67	-3346.36	-103.74	-53.24
2	OpenMM	386.61	375.45	454.22	-2936.22	-87.27	-65.96
	GROMACS	386.61	375.44	454.22	-2936.22	-87.26	-65.95
3	OpenMM	444.53	384.39	400.96	-2925.66	-109.99	-62.71
	GROMACS	444.53	384.39	400.96	-2925.66	-109.98	-62.70
4	OpenMM	387.01	384.20	422.79	-2877.72	-96.31	-53.14
	GROMACS	387.01	384.20	422.79	-2877.72	-96.30	-53.13
5	OpenMM	440.40	383.35	450.34	-2934.92	-88.74	-59.70
	GROMACS	440.40	383.35	450.34	-2934.92	-88.73	-59.70
6	OpenMM	428.49	352.92	445.23	-2913.69	-90.77	-78.06
	GROMACS	428.49	352.92	445.23	-2913.69	-90.77	-78.06
7	OpenMM	473.75	439.53	480.74	-2915.47	-128.57	-54.99
	GROMACS	473.75	439.53	480.74	-2915.47	-128.56	-54.98
8	OpenMM	433.29	375.09	427.08	-2915.08	-127.77	-65.20
	GROMACS	433.29	375.09	427.08	-2915.08	-127.77	-65.19
9	OpenMM	423.03	382.15	455.44	-2982.15	-99.48	-84.06
	GROMACS	423.03	382.15	455.44	-2982.15	-99.48	-84.05
10	OpenMM	452.80	417.20	494.68	-2896.97	-126.01	-85.76
	GROMACS	452.80	417.20	494.68	-2896.97	-126.00	-85.76

Table K: Comparison of potential energies computed with OpenMM and GROMACS using MOFF for proteins and MRG for DNA for ten configurations of HP1α dimer bound to a dsDNA. The energy unit is kJ/mol. See text *Section: Validating the force field implementation in OpenMM* for simulation details.

		Protein				DNA			
Frame ID	Software	Bonds	Angles	Dihedrals	Native pairs	Bonds & fan bonds	Angles	Contacts	Electrostatics
1	OpenMM	5.08	2.70	2.33	-3346.29	98.90	3.21	-106.02	759.21
	GROMACS	5.08	2.70	2.33	-3346.29	98.90	3.21	-106.02	759.04
2	OpenMM	420.00	344.70	459.40	-2940.25	1264.66	411.22	-140.32	686.32
	GROMACS	420.00	344.70	459.40	-2940.25	1264.65	411.22	-140.31	686.27
3	OpenMM	404.52	387.80	480.73	-2913.34	1341.51	438.40	-161.86	673.97
	GROMACS	404.52	387.80	480.73	-2913.34	1341.51	438.40	-161.85	673.91
4	OpenMM	418.64	416.13	472.84	-2878.63	1352.10	416.04	-137.46	633.35
	GROMACS	418.64	416.13	472.84	-2878.63	1352.10	416.04	-137.45	633.32
5	OpenMM	437.58	376.80	452.27	-2869.10	1387.26	413.92	-119.47	665.30
	GROMACS	437.58	376.80	452.27	-2869.10	1387.26	413.92	-119.47	665.26
6	OpenMM	425.41	416.51	502.90	-2874.56	1375.09	390.28	-108.54	600.42
	GROMACS	425.41	416.51	502.90	-2874.56	1375.09	390.28	-108.53	600.37
7	OpenMM	415.22	385.06	466.07	-2922.99	1292.21	390.08	-144.63	641.36
	GROMACS	415.22	385.06	466.07	-2922.99	1292.21	390.08	-144.62	641.30
8	OpenMM	427.79	401.11	488.72	-2922.98	1284.19	393.42	-160.31	595.48
	GROMACS	427.79	401.11	488.72	-2922.98	1284.19	393.42	-160.30	595.43
9	OpenMM	423.46	397.75	537.37	-2917.23	1376.37	381.50	-142.74	570.76
	GROMACS	423.46	397.75	537.37	-2917.23	1376.37	381.50	-142.73	570.71
10	OpenMM	391.07	332.69	509.94	-2903.83	1303.68	410.59	-162.62	567.65
	GROMACS	391.07	332.69	509.94	-2903.83	1303.68	410.59	-162.61	567.60

Table L: Comparison of potential energies computed with OpenMM and HOOMD-Blue using HPS with Urry or KR scales for ten configurations of protein DDX4. The energy unit is kJ/mol. See text *Section: Validating the force field implementation in OpenMM* for simulation details.

Frame ID	Software	Bonds	Contacts (Urry)	Contacts (KR)	Electrostatics
1	OpenMM	399.36	-62.28	-76.41	12.69
	HOOMD-Blue	399.36	-62.28	-76.41	12.69
2	OpenMM	333.40	-67.89	-83.48	13.89
	HOOMD-Blue	333.40	-67.89	-83.48	13.89
3	OpenMM	314.07	-66.08	-82.68	12.95
	HOOMD-Blue	314.07	-66.08	-82.68	12.95
4	OpenMM	341.34	-68.28	-86.45	13.69
	HOOMD-Blue	341.34	-68.28	-86.45	13.69
5	OpenMM	346.92	-68.09	-85.46	14.10
	HOOMD-Blue	346.92	-68.09	-85.46	14.10
6	OpenMM	342.02	-74.57	-94.67	13.08
	HOOMD-Blue	342.02	-74.56	-94.67	13.08
7	OpenMM	252.22	-73.01	-93.11	15.66
	HOOMD-Blue	252.22	-73.01	-93.11	15.66
8	OpenMM	296.98	-63.08	-84.78	11.16
	HOOMD-Blue	296.98	-63.08	-84.78	11.16
9	OpenMM	338.76	-68.30	-88.34	12.56
	HOOMD-Blue	338.76	-68.30	-88.34	12.56
10	OpenMM	386.13	-72.54	-94.43	13.99
	HOOMD-Blue	386.13	-72.54	-94.43	13.99

Table M: Comparison of potential energies computed with OpenMM and LAMMPS using Mpipi force field for a polyR+polyK+polyU system. The system consists of a chain of 10 arginines, a chain of 10 lysines, and 2 individual chains of 10 uracils. The energy unit is kJ/mol. See text *Section: Validating the force field implementation in OpenMM* for simulation details.

Frame ID	Software	Bonds	Contacts	Electrostatics
1	OpenMM	0.07	-12.49	19.22
	LAMMPS	0.07	-12.49	19.22
2	OpenMM	35.10	-22.14	12.26
	LAMMPS	35.10	-22.14	12.26
3	OpenMM	32.01	-37.80	12.77
	LAMMPS	32.01	-37.80	12.77
4	OpenMM	24.04	-62.50	6.19
	LAMMPS	24.04	-62.49	6.19
5	OpenMM	51.21	-29.63	14.43
	LAMMPS	51.20	-29.63	14.43
6	OpenMM	52.29	-33.85	11.85
	LAMMPS	52.29	-33.85	11.85
7	OpenMM	56.81	-37.85	2.63
	LAMMPS	56.81	-37.85	2.63
8	OpenMM	50.78	-28.42	11.88
	LAMMPS	50.78	-28.42	11.88
9	OpenMM	69.27	-72.28	2.20
	LAMMPS	69.27	-72.28	2.20
10	OpenMM	55.98	-36.91	9.33
	LAMMPS	55.98	-36.91	9.33

Table N: The coexistence concentrations of HP1α and HP1β dimers measured by slab simulations with MOFF at different temperatures below the critical temperature. The cutoff distance for searching the largest cluster is 5 nm.

Protein	T (K)	ρ_L (mg/mL)	ρ_H (mg/mL)
HP1α dimer	260	0.02	387.37
	270	0.16	327.11
	280	0.51	305.77
	290	2.51	242.44
	300	13.45	207.17
HP1β dimer	210	0.00	347.36
	220	0.82	310.57
	230	2.31	252.44
	240	7.64	199.42

Table O: The coexistence concentrations of HP1α and HP1β dimers measured by slab simulation with MOFF. The concentrations were similarly determined as those shown in Table N but the cutoff distance for searching the largest cluster set as 8 instead of 5 nm. The results are almost identical to the ones shown in Table N, supporting the robustness of phase diagrams with respect to the cutoff distance used for protein clustering.

Protein	T (K)	ρ_{L} (mg/mL)	ρ_{H} (mg/mL)
HP1α dimer	260	0.02	387.37
	270	0.16	327.11
	280	0.51	305.77
	290	2.51	242.44
	300	13.45	207.17
HP1β dimer	210	0.00	347.36
	220	0.82	310.58
	230	2.31	252.54
	240	7.63	199.00

Table P: The coexistence concentrations of FUS LC and DDX4 proteins measured by performing slab simulations with HPS model Urry scale and the optimal parameter set ($\mu = \mu_{\mathrm{Urry}}^{\mathrm{opt}} = 1$ and $\Delta = \Delta_{\mathrm{Urry}}^{\mathrm{opt}} = 0.08$) at different temperatures below the critical temperature. The cutoff distance for searching the largest cluster is 5 nm.

Protein	T (K)	ρ_{L} (mg/mL)	ρ_{H} (mg/mL)
FUS LC	260	0.00	695.41
	270	0.00	653.23
	280	0.01	610.82
	290	0.28	569.50
	300	0.28	525.02
	310	1.25	480.18
	320	4.96	425.01
	330	14.57	361.59
DDX4	260	0.00	560.53
	270	0.00	514.19
	280	0.10	474.87
	290	0.57	431.43
	300	1.89	379.44
	310	7.69	326.18
	320	47.67	271.05

Reference

1. Latham, A. P.; Zhang, B. Consistent force field captures homologue-resolved hp1 phase separation. *Journal of chemical theory and computation* **2021**, *17*, 3134–3144.

2. Dignon, G. L.; Zheng, W.; Kim, Y. C.; Best, R. B.; Mittal, J. Sequence determinants of protein phase behavior from a coarse-grained model. *PLoS computational biology* **2018**, *14*, e1005941.

3. Regy, R. M.; Thompson, J.; Kim, Y. C.; Mittal, J. Improved coarse-grained model for studying sequence dependent phase separation of disordered proteins. *Protein Science* **2021**, *30*, 1371–1379.

4. Ashbaugh, H. S.; Hatch, H. W. Natively unfolded protein stability as a coil-to-globule transition in charge/hydropathy space. *Journal of the American Chemical Society* **2008**, *130*, 9536–9542.

5. Kapcha, L. H.; Rossky, P. J. A simple atomic-level hydrophobicity scale reveals protein interfacial structure. *Journal of molecular biology* **2014**, *426*, 484–498.

6. Urry, D. W.; Gowda, D. C.; Parker, T. M.; Luan, C.-H.; Reid, M. C.; Harris, C. M.; Pattanaik, A.; Harris, R. D. Hydrophobicity scale for proteins based on inverse temperature transitions. *Biopolymers: Original Research on Biomolecules* **1992**, *32*, 1243–1250.

7. Savelyev, A.; Papoian, G. A. Chemically accurate coarse graining of double-stranded DNA. *Proceedings of the National Academy of Sciences* **2010**, *107*, 20340–20345.

8. Latham, A. P.; Zhang, B. On the stability and layered organization of protein-DNA condensates. *Biophysical Journal* **2022**, *121*, 1727–1737.

9. Joseph, J. A.; Reinhardt, A.; Aguirre, A.; Chew, P. Y.; Russell, K. O.; Espinosa, J. R.; Garaizar, A.; Collepardo-Guevara, R. Physics-driven coarse-grained model for biomolec-

ular phase separation with near-quantitative accuracy. *Nature Computational Science* **2021**, *1*, 732–743.

10. Wang, X.; Ramírez-Hinestrosa, S.; Dobnikar, J.; Frenkel, D. The Lennard-Jones potential: when (not) to use it. *Physical Chemistry Chemical Physics* **2020**, *22*, 10624–10633.

11. Noel, J. K.; Whitford, P. C.; Onuchic, J. N. The shadow map: a general contact definition for capturing the dynamics of biomolecular folding and function. *The Journal of Physical Chemistry B* **2012**, *116*, 8692–8702.

12. Noel, J. K.; Levi, M.; Raghunathan, M.; Lammert, H.; Hayes, R. L.; Onuchic, J. N.; Whitford, P. C. SMOG 2: a versatile software package for generating structure-based models. *PLoS computational biology* **2016**, *12*, e1004794.

13. McGibbon, R. T.; Beauchamp, K. A.; Harrigan, M. P.; Klein, C.; Swails, J. M.; Hernández, C. X.; Schwantes, C. R.; Wang, L.-P.; Lane, T. J.; Pande, V. S. MDTraj: a modern open library for the analysis of molecular dynamics trajectories. *Biophysical journal* **2015**, *109*, 1528–1532.

14. Michaud-Agrawal, N.; Denning, E. J.; Woolf, T. B.; Beckstein, O. MDAnalysis: a toolkit for the analysis of molecular dynamics simulations. *Journal of computational chemistry* **2011**, *32*, 2319–2327.

15. Gowers, R. J.; Linke, M.; Barnoud, J.; Reddy, T. J.; Melo, M. N.; Seyler, S. L.; Domanski, J.; Dotson, D. L.; Buchoux, S.; Kenney, I. M., et al. MDAnalysis: a Python package for the rapid analysis of molecular dynamics simulations. Proceedings of the 15th python in science conference. 2016; p 105.

16. Sugita, Y.; Okamoto, Y. Replica-exchange molecular dynamics method for protein folding. *Chemical physics letters* **1999**, *314*, 141–151.

17. Li, Y.; Zhang, Y. REMO: A new protocol to refine full atomic protein models from C-alpha traces by optimizing hydrogen-bonding networks. *Proteins: Structure, Function and Bioinformatics* **2009**, *76*, 665–676.

18. Bussi, G.; Donadio, D.; Parrinello, M. Canonical sampling through velocity rescaling. *The Journal of chemical physics* **2007**, *126*, 014101.

19. Källberg, M.; Wang, H.; Wang, S.; Peng, J.; Wang, Z.; Lu, H.; Xu, J. Template-based protein structure modeling using the RaptorX web server. *Nature protocols* **2012**, *7*, 1511–1522.

20. Larson, A. G.; Elnatan, D.; Keenen, M. M.; Trnka, M. J.; Johnston, J. B.; Burlingame, A. L.; Agard, D. A.; Redding, S.; Narlikar, G. J. Liquid droplet formation by HP1α suggests a role for phase separation in heterochromatin. *Nature* **2017**, *547*, 236–240.

21. Promoting transparency and reproducibility in enhanced molecular simulations. *Nature methods* **2019**, *16*, 670–673.
